# Development and Validation of a Simulation Framework for Acoustic Intensity Measurements Using Dual-Microphone Probes Under Moving Source and Probe Conditions

**DOI:** 10.3390/s26196233

**Published:** 2026-09-30

**Authors:** Raúl Martín Ferrer, Guillermo Palacios-Navarro, Pedro Ramos Lorente

**Affiliations:** 1 Department of Design and Manufacturing Engineering, University of Zaragoza, 44003 Teruel, Spain; raul.martin@unizar.es; 2 Department of Electronic Engineering and Communications, University of Zaragoza, 44003 Teruel, Spain; guillermo.palacios@unizar.es

**Keywords:** sound intensity, simulation tool, intensity probe, retarded time

## Abstract

**Highlights:**

**What are the main findings?**

- An acoustic propagation simulation framework for acoustic intensity measurement using dual-microphone probes under arbitrary source and probe motion has been developed.
- The simulator incorporates retarded-time computation, trajectory and signal interpolation, and Doppler-related effects within a unified framework showing excellent agreement with theoretical predictions derived from acoustic propagation theory.

**What are the implications of the main findings?**

- The proposed simulator provides a flexible and cost-effective platform for designing, verifying the consistency, and optimizing acoustic intensity measurement systems before conducting experimental campaigns.
- The developed framework enables the systematic evaluation of factors such as probe motion, orientation, and signal-processing algorithms, supporting future advances in sound-power determination and source-localization techniques.

**Abstract:**

This work presents a comprehensive physics-based simulation framework designed to model and assess the performance of acoustic intensity dual-microphone probes under dynamic conditions. The measured quantity, acoustic intensity, is a vector representing the net flow of sound energy through a unit area, allowing for the estimation not only of the amount of energy transmitted but also its direction. This characteristic is of great interest for locating sound sources and evaluating the acoustic power radiated by a source enclosed within a bounding surface. To analyze the quality of the intensity determination, an acoustic propagation model was developed in a MATLAB R2025a environment that allows for the simulation of the generation, three-dimensional propagation, and measurement of sound signals under free-field conditions. The model incorporates moving sources and sensors, precise calculation of propagation delays, and simulation of triaxial probes, also allowing for sensor translations and rotations. From the simulated pressure signals, intensity estimates are obtained using time and frequency-domain methods, and the quality of the estimates is evaluated using error metrics against known geometric references. The results demonstrate the simulator’s ability to generate coherent and controlled datasets and highlight its usefulness as a tool for comparative analysis of intensity estimation methods and for predicting the performance of real probes in dynamic scenarios.

## 1. Introduction

Sound power, or acoustic power, is the rate at which a sound source emits energy in the form of acoustic waves in all directions per unit time, providing an objective descriptor of the noise generated by the source. It is a scalar quantity, independent of direction, that allows different noise sources to be compared reliably because it measures the total acoustic energy radiated per second, expressed in watts (W).

Sound pressure, in contrast, is the instantaneous variation in pressure relative to atmospheric pressure caused by sound waves at a given point in space. It is also a scalar quantity, expressed in pascals (Pa), and is directly perceived as the loudness level of a sound. However, its value depends strongly on the surrounding environment, since reflections, absorption and room geometry can amplify or attenuate the pressure measured at a specific location. Acoustic power can only be related to sound pressure under parti-cular conditions, such as those found in controlled environments like anechoic chambers.

Sound intensity, on the other hand, is a vector quantity defined as the net flow of acoustic energy passing through a unit area in a direction normal to that surface, expressed in watts per square meter (W/m^2^). Sound intensity provides information not only about the amount of energy being transmitted but also about its direction, making it a powerful tool for noise-source localization and for evaluating the acoustic energy crossing a closed surface. Unlike sound pressure, which only describes the amplitude of the wave at a single point, sound intensity reflects the direction of the effective transport of acoustic energy in space. Measuring sound intensity is, however, more challenging because it requires the determination of acoustic particle velocity, whereas sound pressure can be measured more easily and with less expensive instrumentation. Nevertheless, the main advantage of sound intensity measurements is that they allow the sound power of a source to be estimated even in environments where multiple noise sources coexist, something that cannot be achieved using sound pressure measurements alone.

Sound power measurements are performed to quantify and compare the noise emitted by different sound sources. Traditionally, the estimation of sound power has relied on sound pressure measurements. However, these methods become impractical when the source cannot be isolated in a controlled environment or when other noise sources cannot be eliminated during the measurement process. For this reason, sound-power determination methods based on sound intensity measurements were developed.

The development of acoustic intensity measurement has been the result of more than a century of continuous advances in acoustic theory, sensing technologies, instrumentation and signal processing. Rather than representing isolated contributions, these developments constitute successive steps toward increasingly accurate, reliable and practical measurement systems. To provide the necessary context for the present work, the historical evolution of acoustic intensity measurement is reviewed through four major stages: the origins of acoustic intensity measurement, the transition to dual-microphone intensity probes, the improvement of measurement accuracy through calibration and standardization, and the recent emergence of simulation-based methodologies for the analysis and optimization of acoustic intensity measurements.

Origins of acoustic intensity measurement: Following the early theoretical foundations of acoustics, Lord Rayleigh described in [1] the design and operation of an experimental device, known as the Rayleigh disk, for measuring the intensity of airborne vibrations. This device is considered the first practical attempt to measure acoustic intensity and established the conceptual foundations for the future development of acoustic intensity measurement instruments. It demonstrated that sound intensity could be estimated from the interaction between a mechanical object (the disk) and the oscillatory airflow associated with a sound wave. However, the Rayleigh disk was extremely sensitive to non-acoustic air currents, such as drafts and breezes, which produced erroneous readings and limited its use outside controlled environments. Consequently, it was never adopted as a practical instrument for acoustic intensity measurements.

At the beginning of the twentieth century, advances in electronics, and particularly the invention of the condenser microphone by Edward C. Wente at Bell Laboratories [2], enabled significant progress in the measurement of acoustic parameters.

Harry F. Olson patented in 1932 the first acoustic intensity meter that was truly functional and suitable for practical applications [3]. The instrument was designed to measure both the direction and the flow of acoustic energy, even in non-anechoic sound fields.

Clapp and Firestone [4] describe the construction and validation of the first acoustic wattmeter, an instrument capable of directly measuring acoustic intensity as the product of sound pressure and particle velocity at a single point. The system combines a pressure microphone (crystal microphone) and a particle-velocity microphone mounted together, allowing pressure and velocity to be measured independently; the product of these two quantities yields the acoustic energy flux. This instrument established the conceptual and experimental foundations that would later evolve into the modern two-microphone acoustic intensity probes developed several decades afterward.

In this contribution [5], presented at the 87th Meeting of the Acoustical Society of America, Harry F. Olson described the field-type acoustic wattmeter, developed from his original 1932 patent. The instrument combines a pressure microphone and a particle-velocity microphone to measure acoustic energy flow directly in real-world environments. Olson proposed solid-state electronics to process the signals and compute acoustic intensity as the product of instantaneous pressure and particle velocity. The design emphasizes practical field applications rather than laboratory-only use, including the measurement of loudspeaker sound power, in situ assessment of machinery noise, evaluation of the acoustic absorption of walls, ceilings, and floors, and characterization of sound fields within enclosures. Olson’s work is particularly significant because it helped standardize the application of acoustic-intensity measurement techniques in practical environments, bridging the gap between conceptual methods and robust solutions for the audio industry and acoustic engineering. This development ultimately led to the first commercial portable acoustic intensity meters in the 1970s, facilitating direct sound-power measurements as the first international standards for sound and acoustic intensity were being established.

In 1955, Baker [6] presented one of the first practical prototypes of an acoustic intensity meter, developed at the Massachusetts Institute of Technology (MIT). Unlike later approaches based on pairs of pressure microphones, Baker’s instrument combined a crystal microphone acting as a pressure transducer with a hot-wire anemometer designed to measure particle velocity.

Schultz [7] proposed in 1956 an acoustic wattmeter prototype capable of directly measuring acoustic intensity as the time-averaged product of sound pressure and particle velocity. The instrument provided continuous data through an analog pointer display, offering a dynamic range of 50 dB and an operating frequency range extending up to 10 kHz. The principal innovation of Schultz’s work was the practical implementation of an acoustic intensity measurement system with direct, real-time output obtained from an analog instrument that combined pressure and velocity signals instantaneously. This represented a significant advance over earlier systems (e.g., refs. [4,5]), which generally required laboratory conditions or external signal processing. Furthermore, the compact construction of the microphone pair reduced errors associated with microphone spacing, laying the groundwork for the development of modern two-microphone acoustic intensity meters.

In 1973, Burger et al. [8] presented an experimental system for measuring acoustic intensity with the aim of determining the sound power radiated by a source. Their approach was also based on calculating acoustic intensity through the multiplication of the signals from a pressure microphone and a particle-velocity microphone. The resulting intensity function was subsequently integrated over a closed surface to determine the total sound power emitted by the source. Their study demonstrated that intensity measurements are largely independent of the characteristics of the acoustic field, whether free-field, reverberant, or semi-reverberant. The authors showed that the technique enables highly accurate in situ measurements and described a combined microphone system capable of measuring acoustic intensity over the frequency range from 50 Hz to 12 kHz. The importance of this work lies in its experimental demonstration that sound-power measurements based on acoustic intensity are less sensitive to environmental effects than conventional pressure-based measurements. As a result, it provided a solid foundation for the development of acoustic-intensity measurement techniques suitable for real-world applications.

Transition to dual-microphone intensity probes: The developments described above established the experimental foundations of acoustic intensity measurement by demonstrating that acoustic energy flow could be measured directly through pressure and particle-velocity sensing. Nevertheless, the practical implementation of these instruments remained complex because of the difficulty of accurately measuring particle velocity. This limitation motivated the development of a new generation of techniques based exclusively on pressure measurements, giving rise to the modern dual-microphone approach that forms the basis of contemporary acoustic intensity probes.

In 1977, Pavić [9] introduced a method for measuring acoustic intensity in non-dispersive media using only two omnidirectional microphones. He demonstrated that the components of the acoustic intensity vector can be determined by measuring sound pressure and estimating particle velocity from the pressure gradient. The paper presents a detailed analysis of the errors associated with microphone spacing, phase mismatch, averaging time, and noise, and provides explicit expressions for optimizing the microphone separation as a function of frequency in order to minimize relative errors. Pavić’s work established a rigorous formulation of the two-microphone method, providing a solid theoretical framework for quantifying measurement uncertainty. The study also demonstrated experimentally that reliable intensity measurements require both a carefully selected microphone spacing and adequate phase compensation. In addition, it proposed a frequency-band measurement approach that extends the applicability of the method to lower frequencies, thereby significantly enhancing its practical usefulness in acoustic intensity measurements.

Fahy’s work [10] is widely regarded as the classical reference text in the field of acoustic intensity measurement, providing both the theoretical foundations and the practical methodologies of sound-intensity techniques. In addition, Jacobsen and Juhl [11] offer a comprehensive treatment of acoustic intensity measurement and its relationship to the underlying principles of acoustic theory. Earlier, in 1977, Fahy [12] proposed a two-microphone method for estimating acoustic intensity, contributing significantly to the development and practical implementation of modern *p*–*p* acoustic intensity probes.

Alternative measurement approaches have also been developed to obtain particle-velocity information without relying on the pressure-gradient approximation used in conventional *p*–*p* probes. In particular, the Microflown sensor enables direct measurement of acoustic particle velocity and, when combined with a pressure microphone in a *p*–*u* probe, provides an alternative approach to sound-intensity measurement. Jacobsen and de Bree [13] compared the *p–p* and *p–u* principles, showing that they exhibit different sources of measurement error and highlighting the importance of phase calibration in *p*–*u* probes, particularly under highly reactive near-field conditions. Microflown sensors have also been employed in recent architectural-acoustics applications, such as the characterization of existing theatre seating at the Royal Festival Hall, with the measured data subsequently incorporated into a geometrical-acoustics model [14]. Other non-contact approaches include Laser Doppler Vibrometry (LDV), which has been applied to the in situ determination of normal acoustic impedance and sound absorption by combining surface-vibration velocity measurements with acoustic pressure measurements. Saccenti et al. [15] demonstrated this approach on historical materials for which contact or sample extraction was undesirable. These techniques complement conventional dual-microphone probes and illustrate the broader range of sensing strategies available for acoustic characterization.

Improving accuracy, calibration and standardization: Once the dual-microphone principle had become established, research efforts progressively shifted from the formulation of acoustic intensity estimation methods toward improving their measurement accuracy, robustness and practical applicability. Particular attention was devoted to reducing the influence of phase mismatch, microphone positioning errors and probe calibration, while international standards and industrial applications promoted the widespread adoption of acoustic intensity measurements. These developments transformed acoustic intensity probes from laboratory instruments into reliable tools for engineering practice. Despite these advances, the systematic evaluation of probe performance under controlled and dynamic measurement conditions still remains a challenging task.

The contributions of Yanagisawa and Koike [16] addresses two of the major sources of error in acoustic intensity measurements based on the two-microphone method: the intrinsic phase mismatch between microphones, which introduces particularly severe errors at low frequencies, and positioning errors that occur when the axis defined by the microphone pair is not perfectly aligned with the direction of sound propagation. To overcome these limitations, the authors proposed a technique based on rotating the microphone pair around the measurement point within specific planes and mathematically processing the acquired signals by integrating them over the rotation angles. By averaging the measurements over a complete rotation cycle, the procedure effectively cancels both phase-mismatch and positioning errors, enabling the three components of the acoustic intensity vector to be determined with high accuracy.

Based on the formulation of acoustic intensity as a function of the imaginary part of the cross-spectrum between the signals measured by two microphones, Chung [17] proposed a circuit-switching technique that effectively compensates for instrumental phase mismatch. The study develops and validates a practical method for acoustic intensity measurement that minimizes the errors introduced by phase differences between the instrumentation channels. This contribution was particularly important because it provided a simple and effective means of improving the accuracy of two-microphone intensity probes without requiring highly complex calibration procedures, thereby enhancing the reliability of acoustic intensity measurements, especially at low frequencies where phase-mismatch errors are most critical.

In his contribution [18], Astrup summarizes fifteen years of professional experience applying acoustic intensity techniques in complex industrial environments, highlighting both their advantages and limitations from a practical perspective. He explains that the scanning method standardized in ISO 9614-2 [19] is generally preferred for in situ measurements because of its efficiency and reduced measurement time, whereas the discrete-point method defined in ISO 9614-1 [20] is better suited to laboratory conditions and detailed analyses. Astrup identifies several key challenges associated with practical acoustic intensity measurements, including the need to vary microphone spacing to cover a wide frequency range, the sensitivity of the technique to air turbulence, and the difficulty of assessing measurement accuracy in real time. One of the most significant contributions of his work is the proposal of specific improvements to instrumentation aimed at enhancing the practical applicability of acoustic intensity measurements, including increased dynamic range, intrinsically safe instrumentation, and real-time quality-control systems capable of verifying measurement accuracy during the measurement process itself.

Continuing the effort to reduce the adverse effects of positioning and phase errors in acoustic intensity measurements, Juhl and Jacobsen [21] demonstrated the importance of properly calibrating intensity probes and improving signal-processing algorithms in order to enhance the accuracy of sound-pressure measurements and, consequently, the reliability of the pressure–intensity index. Their work highlights that careful probe calibration and appropriate signal correction procedures are essential for minimizing systematic measurement errors and improving the overall performance of acoustic intensity measurement systems.

Simulation-based analysis and virtual measurement frameworks: The developments reviewed above have led to highly reliable acoustic intensity probes and well-established measurement procedures. Nevertheless, evaluating the influence of probe geometry, sensor motion, signal-processing algorithms and measurement conditions still relies largely on experimental campaigns, which are often costly, time-consuming and difficult to reproduce under controlled conditions.

Laville and Nicolas [22] developed an early computer simulation of sound power determination based on two-microphone sound intensity measurements. Their approach computed both exact acoustic intensity and the intensity estimated from the pressure signals at two spatially separated microphone positions, allowing the influence of finite-difference, phase-mismatch, and spatial-sampling errors to be investigated under different acoustic-field conditions. This work demonstrated the value of numerical simulation for analysing the accuracy and limitations of dual-microphone intensity measurements. However, the formulation considered stationary source and measurement configurations and did not address the dynamic measurement scenarios associated with moving sources, moving probes, or time-varying probe orientation.

Recent advances in computational acoustics have progressively expanded the role of physics-based simulation beyond the prediction of acoustic fields. Numerical models are now employed not only to reproduce sound propagation, but also to support experimental design, analyze measurement uncertainty, visualize complex acoustic phenomena and estimate acoustic quantities through model-based approaches. Advanced propagation models have been developed to simulate sound fields generated by moving sources in realistic environments, incorporating Doppler effects, complex terrain and impedance boundaries [23]. More recently, physics-based simulations have been integrated with mixed-reality visualization systems, allowing measured and simulated sound intensity distributions to be analyzed simultaneously and facilitating the interpretation of complex acoustic phenomena [24]. In parallel, flexible simulation frameworks have been proposed to generate physically consistent multichannel audio for moving sound sources and arbitrarily configured microphone arrays, supporting the development and evaluation of beamforming, sound-source localization and spatial-audio processing algorithms [25]. Simulation has also been employed to investigate specific measurement issues associated with acoustic intensity acquisition. For example, the influence of microphone-array motion and Doppler effects on sound intensity estimation has been analyzed under controlled measurement conditions, highlighting the impact of probe movement on estimation accuracy [26]. Beyond the simulation of propagation and specific measurement effects, model-based virtual sensing techniques have also been proposed to estimate acoustic quantities at locations where direct measurements are not available. In particular, Van Ophem et al. combined finite-element modelling with Kalman filtering to reconstruct acoustic pressure and sound intensity fields from a limited number of experimental measurements [27]. Although these contributions demonstrate the increasing role of simulation and virtual sensing in modern acoustics, their objectives and capabilities differ from those of the framework proposed in this work. Previous approaches have addressed specific aspects of the problem, including acoustic-field prediction, virtual sensing, multichannel signal generation for moving sources, and the analysis of particular effects on sound-intensity measurements. For example, model-based virtual sensing has been used to estimate acoustic pressure and intensity fields by combining experimental measurements with numerical vibro-acoustic models, whereas other simulation environments generate multichannel microphone signals for moving sound sources and arbitrary microphone-array geometries. Earlier studies have also investigated the numerical simulation of sound-power determination using two-microphone intensity measurements. However, these approaches were developed for different purposes and do not simultaneously address all the capabilities considered here. Table 1 summarizes the main differences between representative previous approaches and the proposed framework.

Despite these advances, important challenges remain in the experimental analysis of acoustic intensity measurement systems. Factors such as source and probe motion, probe orientation, mechanical vibrations, scanning trajectories and signal-processing algorithms frequently interact simultaneously, making their individual effects difficult to isolate through physical experiments alone. Consequently, there is a growing need for physically consistent virtual measurement environments capable of reproducing realistic acoustic intensity measurements under controlled yet highly flexible conditions. Such environments would enable systematic investigations of measurement uncertainty, facilitate the optimization of experimental procedures, and allow repeatable evaluation of new probe configurations and signal-processing strategies before their practical implementation.

To address this need, this paper presents a physics-based virtual acoustic measurement framework specifically designed for acoustic intensity measurements. Rather than simulating only the acoustic field, the proposed framework reproduces the complete acoustic intensity measurement process, from sound propagation and virtual microphone signal generation to acoustic intensity estimation and quantitative performance evaluation. The proposed framework combines an acoustic propagation model with a virtual dual-microphone intensity probe capable of arbitrary translations and rotations, together with both time-domain and frequency-domain intensity estimation methods. By accurately reproducing the acoustic signals received by moving probes from moving sound sources, the framework enables controlled virtual experiments for analyzing, predicting and optimizing the behavior of acoustic intensity measurement systems. Beyond reproducing realistic measurement scenarios, it provides a flexible research environment for investigating phenomena that are difficult to study experimentally (e.g., the influence of probe vibrations) thereby facilitating the design and optimization of future acoustic intensity measurement techniques.

Unlike previous simulation environments, the objective of the proposed framework is not to introduce a new acoustic intensity estimation method, but to provide a physically consistent virtual measurement environment capable of generating the pressure signals required by the most widely used acoustic intensity measurement techniques. For this reason, both time-domain and frequency-domain intensity estimation methods have been incorporated into the framework. Their implementation extends the applicability of the proposed platform to different measurement procedures while providing an additional level of validation by demonstrating that independent processing approaches produce results consistent with the theoretical acoustic field. Furthermore, the modular architecture of the framework facilitates the incorporation, validation against analytical reference solutions and objective comparison of future acoustic intensity estimation techniques, reinforcing its value as a general research platform for the development, evaluation and validation of acoustic intensity measurement methodologies.

Accordingly, the validation of the proposed framework is structured around three research questions: (1) Does the simulated acoustic intensity reproduce the inverse-square dependence on source–probe distance expected under free-field conditions? (2) Does the virtual probe correctly reproduce the combined influence of source–probe distance and measurement geometry on the estimated intensity? (3) Does the simulated probe reproduce the theoretical directional response of an ideal pressure–pressure intensity probe as its orientation changes? In addition, the consistency between the time-domain and frequency-domain intensity estimation methods is evaluated when both are applied to the same simulated pressure signals.

## 2. Materials and Methods

The presented work focuses on the development of an acoustic-signal propagation model implemented in MATLAB R2025a, aimed at predicting the performance and accuracy of sound-intensity measurements obtained using dual-microphone probes. The proposed framework reproduces the generation, propagation and acquisition of acoustic signals under controlled free-field conditions, providing complete knowledge of the acoustic field and its associated intensity vector at every point in space. This information makes it possible to assess the accuracy of different intensity-estimation methods by comparing the estimated values with theoretical reference solutions. In addition, the model supports arbitrary three-dimensional trajectories for both sound sources and sensors, allowing the simulation of static and dynamic measurement scenarios. The resulting environment constitutes a flexible tool for the analysis, numerical assessment and optimization of acoustic intensity measurement techniques under conditions that are difficult to reproduce experimentally.

The proposed framework has been conceived as a general-purpose virtual experimental platform capable of reproducing a broad range of acoustic intensity measurement scenarios involving arbitrary source and probe motion. Although the simulator supports considerably more complex configurations, the present work demonstrates the physical and numerical consistency of the framework through a representative set of controlled experiments specifically selected to verify the physical consistency of the propagation model and the implemented acoustic intensity estimation methods. These assessment scenarios provide the necessary confidence in the framework while illustrating its potential for addressing a much wider range of future research and engineering applications.

### 2.1. Acoustic Propagation Model

This tool has been implemented to simulate the generation, three-dimensional propagation, and measurement of acoustic signals for the purpose of estimating sound intensity. The model allows both sources and sensors to be in motion, which requires precise computation of all propagation delays involved. The number of sources and sensors used in the simulations is enough to reproduce the signals captured by one (or more) triaxial probes consisting of six microphones aligned along three mutually perpendicular axes. The model permits arbitrary translations of both sources and probes in any spatial direction, as well as rotations of the probes about any axis, either fixed or time-varying. Regarding the emitted signals, each source can be assigned either an analytical expression defining the waveform or an externally provided audio file (e.g., in WAV format).

#### 2.1.1. Sound Source Model

The simulation framework models sound sources as omnidirectional point emitters radiating in a homogeneous free-field medium. Under these assumptions, acoustic propagation is governed exclusively by spherical spreading and propagation delay, neglecting reflections, diffraction effects and atmospheric absorption. This simplified scenario provides a controlled environment suitable for validating acoustic intensity estimation methods against theoretical predictions.

#### 2.1.2. Propagation Delay and Retarded Time

A key concept within the model is the propagation delay, which identifies the instant at which a pressure perturbation must have been emitted by the source so as to reach the receiver at a given time and position. This parameter is essential for understanding how acoustic perturbations propagate and how they are perceived by an observer.

Signal attenuation depends not only on the distance between the source (at the retarded time *t_r_*) and the observer (at the current time), but also on a correction factor that accounts for relative motion: attenuation decreases as the source approaches and increases as it recedes.

When solving the nonhomogeneous propagation equation, it must be considered that the simulator operates with discrete-time signals defined at a finite set of points. This discrete nature applies both to the trajectories of sources and sensors and to the time instants at which acoustic signals are defined and subsequently received at the sensor positions. Consequently, the model requires two interpolation stages: (1) trajectory interpolation and (2) acoustic-signal interpolation.

To clarify trajectory interpolation: in the search for the retarded time *t_r_* at which the source must have emitted the perturbation that reaches the sensor at the desired instant, the solution is first bracketed between two sampling instants corresponding to two known probe positions. An interpolation step then provides the precise source position at *t_r_*. As for the interpolation of the acoustic signals, once *t_r_* and the source-to-probe distance *r* are known, the emitted signal—either from an audio file or from an analytical model—can be evaluated at the required retarded time. With *t_r_* and the corresponding emitted signal value determined, the attenuation factor is computed. Accurately determining the source’s velocity vector is crucial, especially when the relative source–probe speed approaches the speed of sound, since this strongly influences the attenuation model. Finally, the simulator applies the principle of superposition: for each microphone of each probe, the pressures contributed by all sources in the acoustic field are summed.

The proposed formulation, already detailed in [28], extends conventional retarded-time approaches beyond analytical constant-velocity motion [25] by allowing arbitrary discretized source and probe trajectories. This extension is not merely a change in trajectory representation: it requires continuously differentiable interpolation, segment-wise numerical determination of the retarded time, and dynamic evaluation of the Doppler correction term from the instantaneous source velocity. Consequently, the proposed framework remains physically consistent for general time-varying motions while preserving the accuracy of the simulated acoustic field.

#### 2.1.3. Source and Receiver Motion

The proposed framework supports arbitrary three-dimensional trajectories for both sound sources and receivers. Their positions are defined as functions of time, allowing the simulation of static configurations as well as dynamic measurement scenarios. The propagation delay is computed considering the instantaneous positions of both elements, enabling the accurate reproduction of Doppler-related effects and time-varying source–receiver geometries.

**Figure 1:**
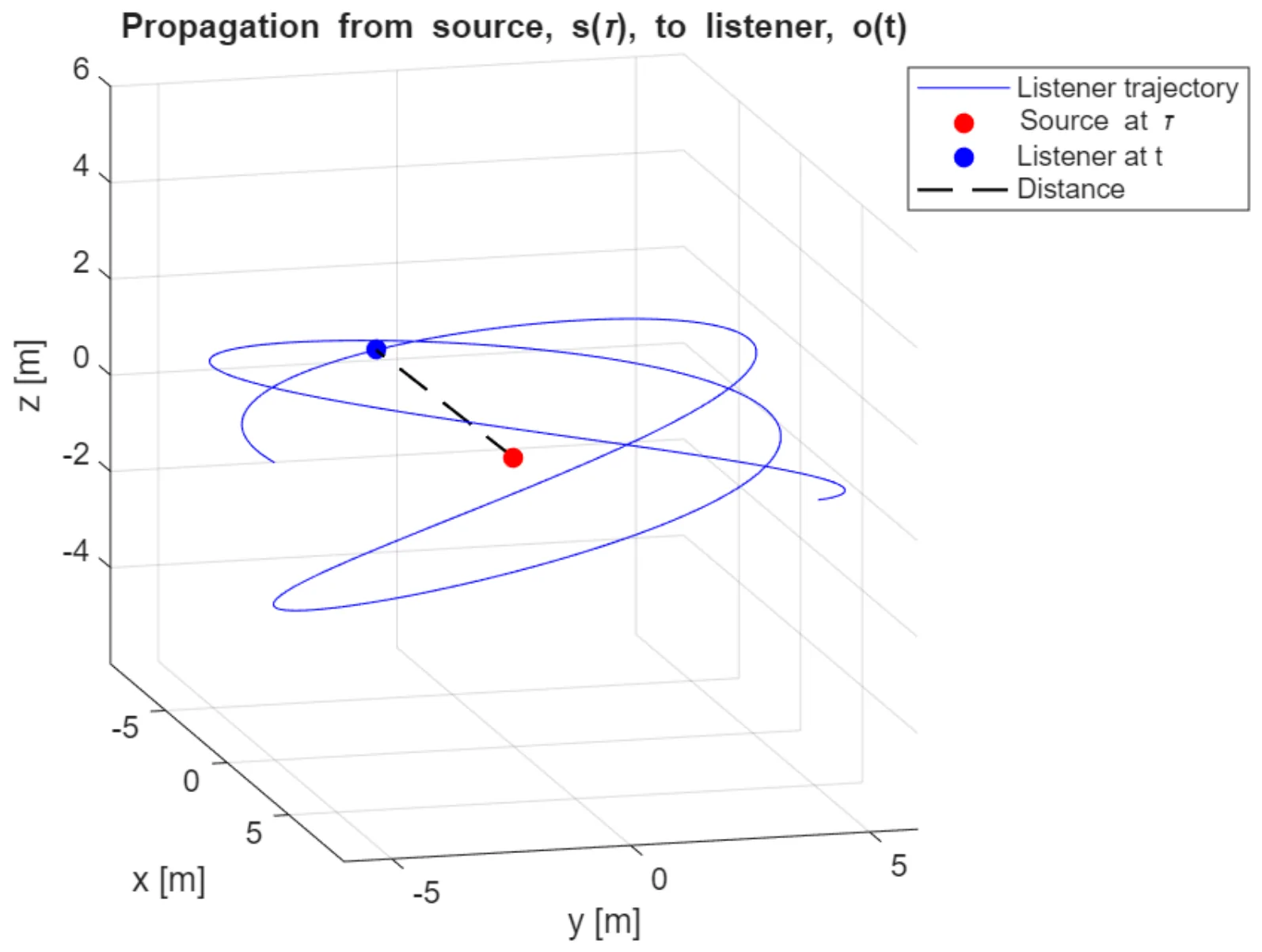
Trajectory of a probe moving around a sound source.

Figure 1 shows a three-dimensional example of a listener moving around a fixed source. The signal emitted by the source consists of three tones, which are perceived with Doppler-like alterations at the listener/probe position.

**Figure 2:**
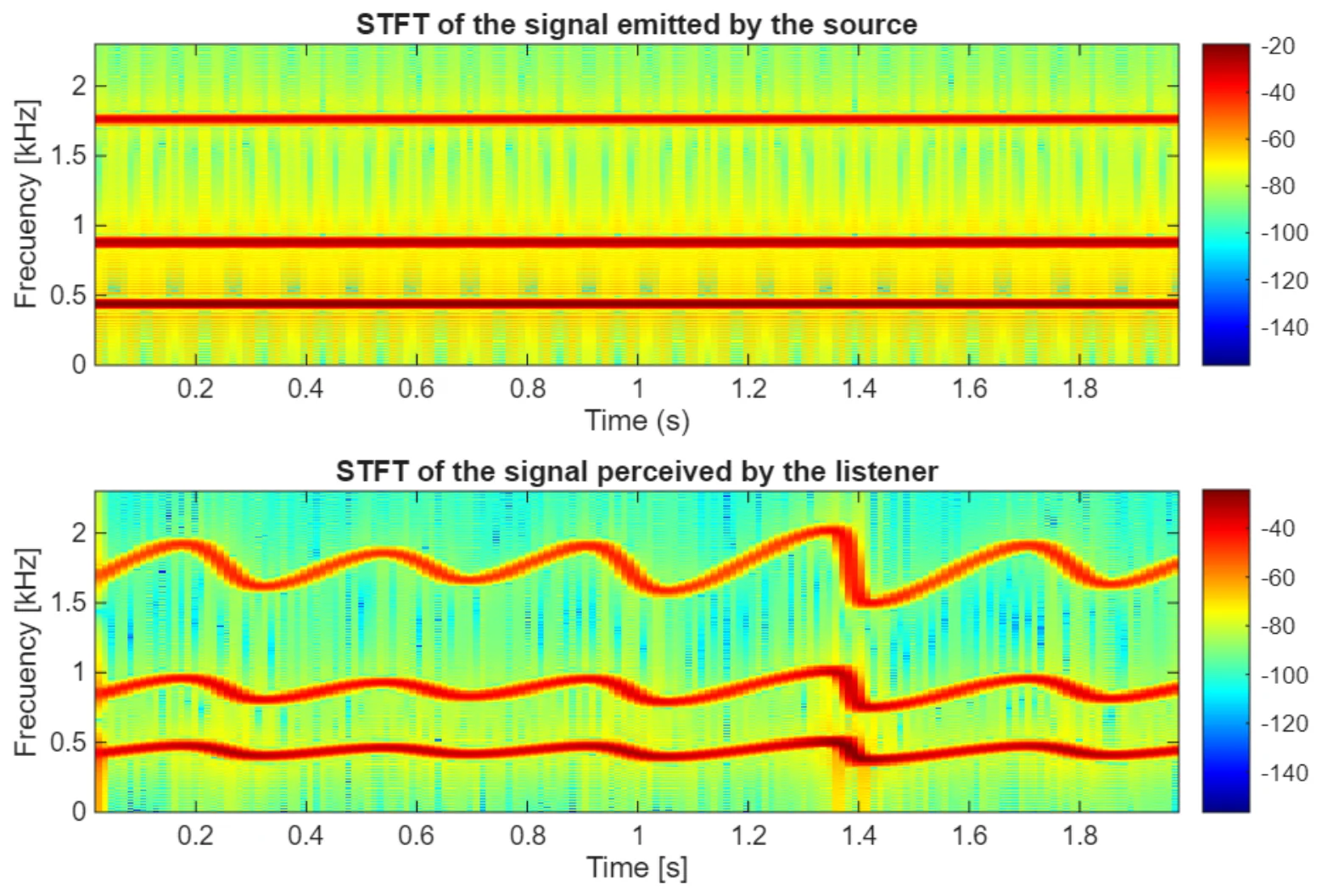
Effect of the source’s movement on the perceived signal.

Figure 2 shows the Short-Time Fourier Transform (STFT) of the emitted and perceived signals.

We therefore have at our disposal a tool capable of accurately determining the sound-pressure levels generated by acoustic sources—which may themselves be in motion—at any point along the trajectory followed by a moving sensor. The key contribution of this tool is that it provides distinguishable values at two points that are very close to each other, enabling the computation of sound intensity from those measurements.

### 2.2. Virtual Acoustic Intensity Probe

#### 2.2.1. Probe Geometry

**Figure 3:**
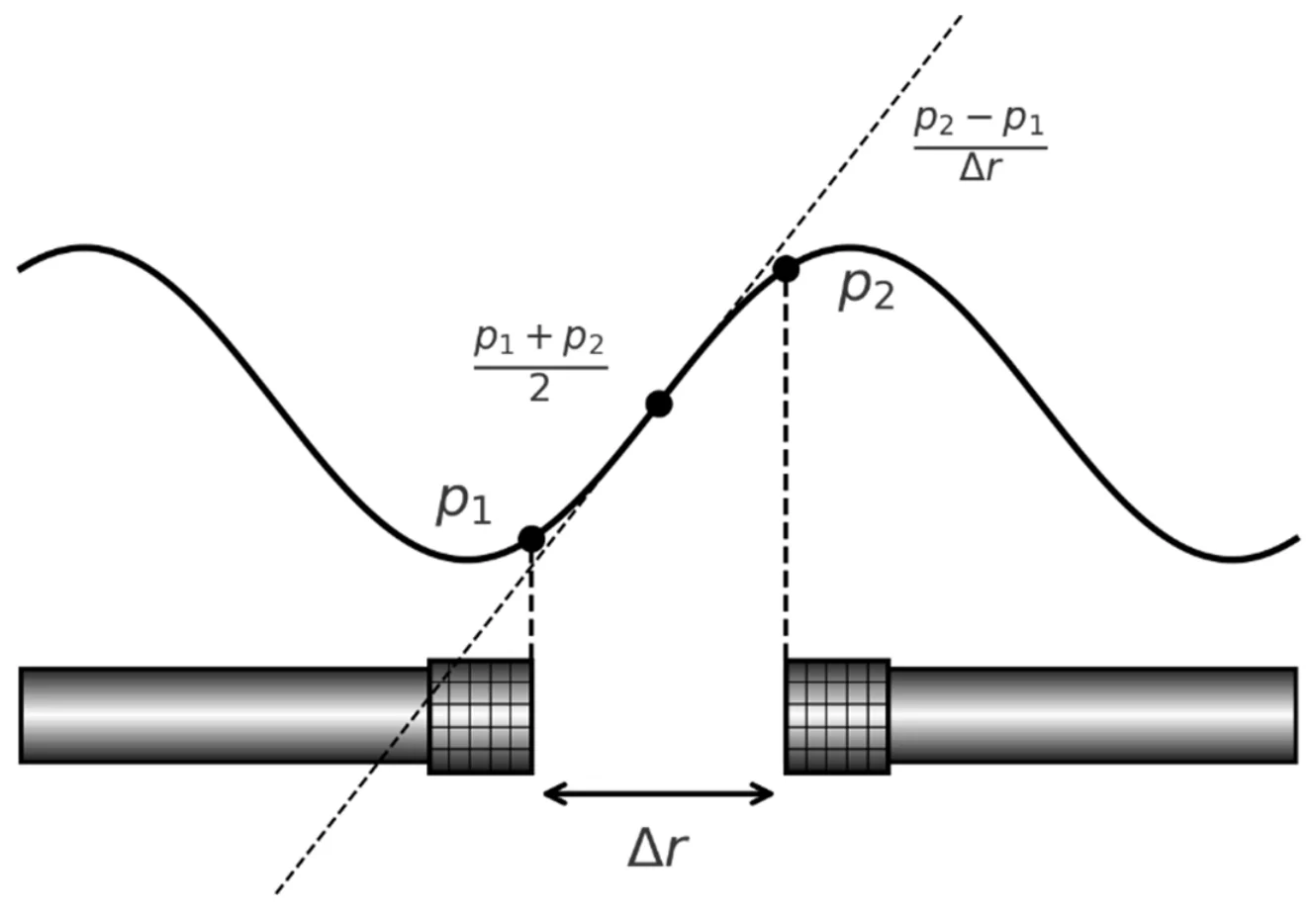
Geometry of the virtual *p*–*p* acoustic intensity probe.

The virtual acoustic intensity probe implemented in the proposed simulation framework is based on the well-established pressure–pressure (*p*–*p*) configuration, consisting of two closely spaced omnidirectional microphones shown in Figure 3. The microphones are positioned along a common measurement axis and separated by a distance ∆*_r_*, which can be selected according to the frequency range under study. The midpoint between both microphones is defined as the probe acoustic center and represents the reference position of the measurement.

The microphone spacing ∆*_r_* represents an important design parameter in a pressure–pressure acoustic intensity probe, since it directly affects both measurement accuracy and the usable frequency range. The pressure-gradient component is estimated from the finite difference between the pressures measured at the two microphone positions. Consequently, a smaller microphone spacing provides a better approximation of the spatial pressure gradient and extends the validity of the measurement toward higher frequencies. However, as the spacing decreases, the pressure difference between the microphones also becomes smaller, increasing the relative influence of microphone phase mismatch and measurement uncertainty, particularly at low frequencies. Conversely, increasing the microphone spacing produces a larger measurable pressure difference and improves low-frequency sensitivity, but the finite-difference approximation becomes inaccurate at lower frequencies as the acoustic wavelength approaches the microphone spacing. Therefore, probe spacing involves a compromise between low-frequency sensitivity and high-frequency accuracy. In the present work, a microphone separation of 12 mm is adopted as a representative value for a conventional dual-microphone intensity probe; its frequency-dependent limitations are explicitly evaluated in the validation experiments presented below.

At each simulation instant, the positions of the two microphones are determined from the probe center coordinates and its spatial orientation. This approach allows the probe to be arbitrarily translated and rotated in three-dimensional space while preserving the relative microphone spacing. Consequently, the framework can reproduce both static measurements and dynamic acquisition scenarios involving moving sensors.

The pressure signals received by the microphones are computed independently using the acoustic propagation model described in Section 2.1 and further detailed in Section 2.3. These signals constitute the primary data used for the subsequent estimation of acoustic intensity through time-domain and frequency-domain processing techniques.

#### 2.2.2. Virtual Microphone Signals

For each simulation time instant, the referred acoustic propagation model is used to determine the pressure signal received at each microphone position. The received pressure depends on the emitted waveform, the time-varying propagation distance between the moving source and microphone, and the corresponding propagation delay. Since the source and the probe may follow arbitrary three-dimensional trajectories, these quantities are continuously updated throughout the simulation.

The pressure signals are generated independently for each microphone, taking into account the exact source-to-receiver geometry at the emission and reception instants. To accurately reproduce dynamic scenarios, the emission time associated with each received sample is obtained from the retarded time formulation. The emitted waveform is subsequently evaluated at the calculated emission instant by interpolation, allowing the simulation of arbitrary source signals and motions.

The resulting microphone signals constitute the fundamental input data for the acoustic intensity estimation procedures. Since both channels are generated from the same physical propagation model, the framework provides complete control over the acoustic field parameters and eliminates the uncertainties commonly associated with experimental measurements, such as sensor mismatch, environmental disturbances or positioning errors.

#### 2.2.3. Probe Orientation and Motion

The proposed simulation framework allows the virtual intensity probe to undergo arbitrary translations and rotations in three-dimensional space. The probe position is defined by the coordinates of its acoustic center, while its orientation is specified through a unit vector aligned with the measurement axis. Consequently, the positions of the individual microphones are dynamically recalculated during the simulation according to the instantaneous probe location and orientation.

This capability enables the reproduction of a wide range of measurement scenarios, including fixed-point measurements, scanning procedures, probe vibration, and dynamic acquisitions involving moving sensors. Since the microphone coordinates vary with time, the source-to-microphone distances and the associated propagation delays are recomputed at each simulation step.

The implementation is not restricted to linear trajectories or constant velocities. Both translational and rotational motions can be defined as arbitrary functions of time, allowing the simulation of complex measurement paths and realistic operating conditions. This flexibility makes the proposed framework suitable for investigating the influence of probe motion on acoustic intensity estimation and for evaluating measurement strategies prior to experimental implementation.

Furthermore, the same formulation can be applied to both single-axis and triaxial probe configurations, making it possible to simulate the acquisition of complete three-dimensional acoustic intensity vector fields.

#### 2.2.4. Extension to Triaxial Probes

**Figure 4:**
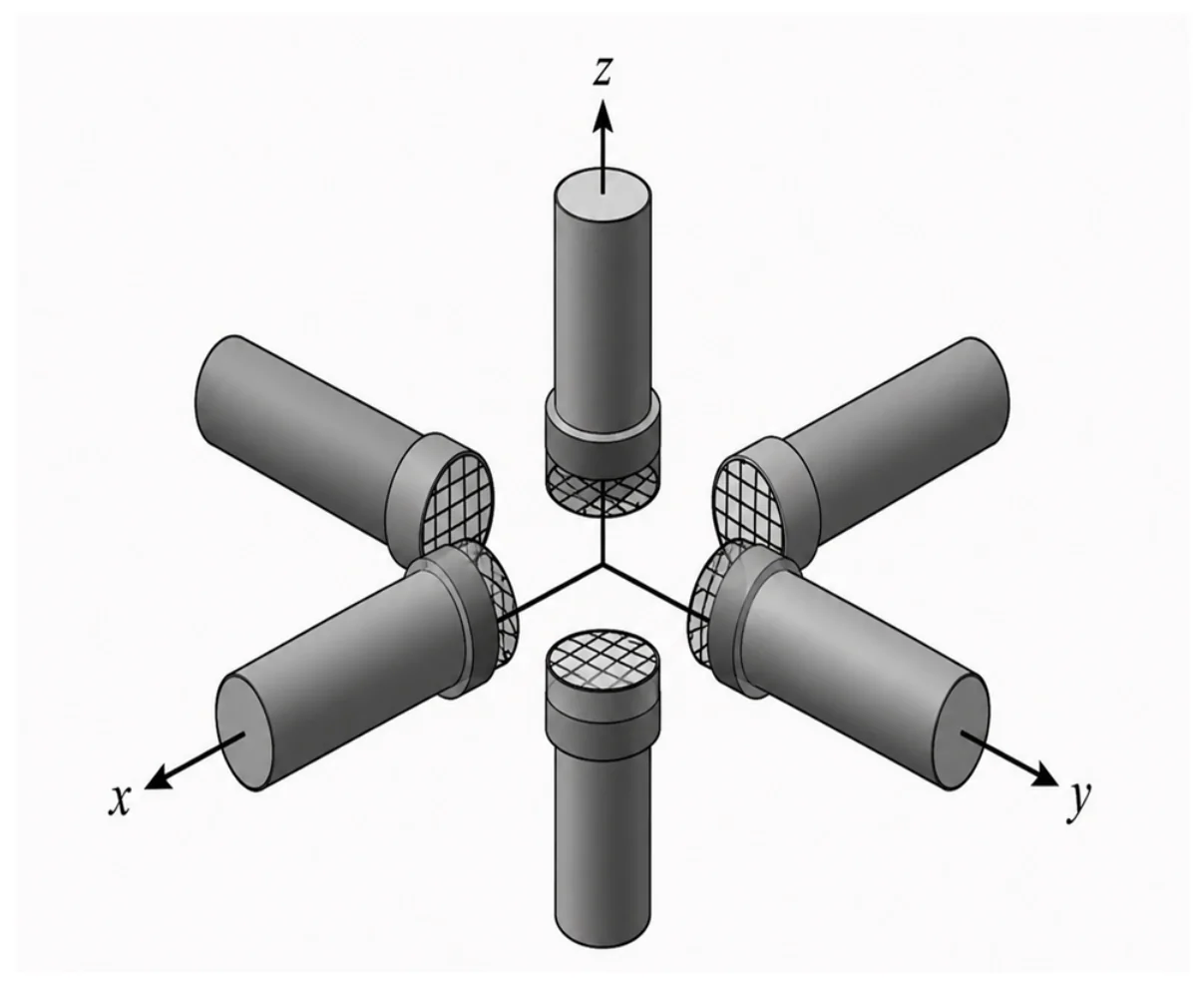
Schematic of a triaxial acoustic probe based on six microphones arranged in opposing pairs along three mutually perpendicular axes.

Although the present work focuses on a single measurement axis, the same formulation can be extended to triaxial configurations composed of three orthogonal microphone pairs (see Figure 4), enabling the estimation of the complete three-dimensional acoustic intensity vector.

### 2.3. Computation of the Received Signals

#### 2.3.1. Retarded Time Computation

In acoustic propagation problems involving moving sources and receivers, the pressure signal received at a given instant is not generated by the source at the same time instant, but rather at an earlier emission time. This delay arises from the finite speed of sound and is commonly described through the concept of retarded time.

Particular attention has been devoted to the accurate determination of the retarded time, since it constitutes the basis for modelling moving sources and receivers and for reproducing Doppler-related phenomena within the simulation environment [28].

Previous work has involved the simulated recreation of a three-dimensional space in which there is an acoustic field generated by different moving sound sources with defined trajectories. The sources emit according to a free-field spherical wave propagation model. In this scenario, it is essential to study both the “retarded (or delay) time” between source and listener and the attenuation of the signal with distance. If source and listener are moving, we must know the distance between the source (at the delay time, when it was emitted) and the listener (at the time of interest), the velocity vector of the source at the delay time and correctly manage the resampling/interpolation of the signals to work on a common time base for source and listener.

**Figure 5:**
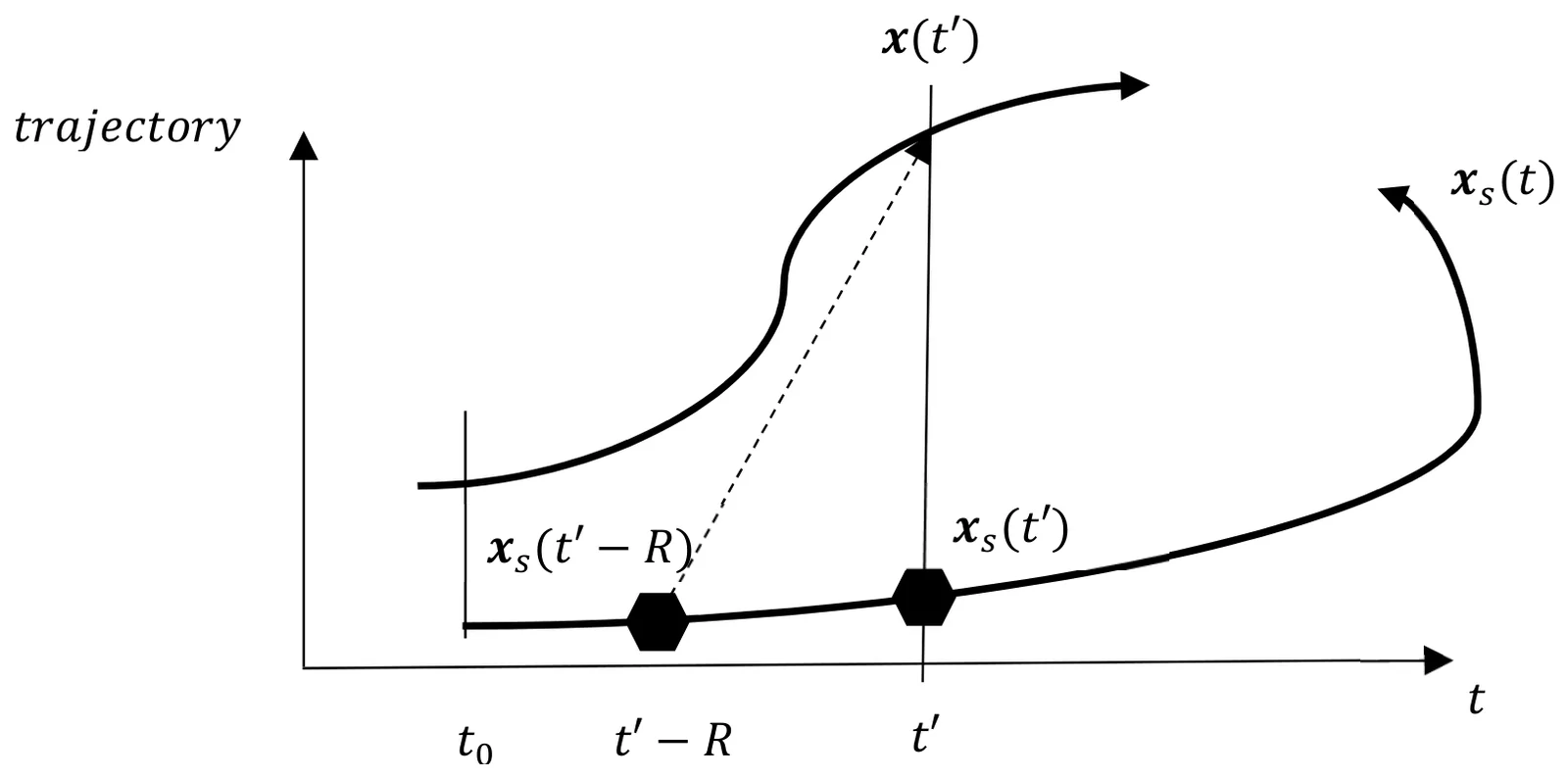
Source and probe trajectories.

Let a sound source and an acoustic probe move according to the 3D trajectories described in Figure 5.

Consider a scenario in which an acoustic probe and a sound source move along independent trajectories. Let ***x***(*t*) denote the trajectory of the probe in three-dimensional space, and let ***x****_s_*(*t*) denote the trajectory of the source. Let *f*(*t*) be the signal emitted by the source at each instant along its trajectory ***x****_s_*(*t*). Let *t′* be an arbitrary time instant and *t*_0_ the initial time at which the source begins its motion.

At the arbitrary instant *t′*, the source is located at the point ***x****_s_*(*t′*) of its trajectory. The aim is to determine the effect of the source, at that instant, on a point of the probe trajectory given by ***x***(*t′*).

The delay time determines the instant at which the source, located at ***x****_s_*(*t′*) emitted a pressure disturbance and this arrives at the receiver located at instant *t′* at position ***x***:

*t_r_*(***x***,***x****_s_*, *t′*) = *t′* − |***x***(*t′*) − ***x****_s_*(*t′* − *R*)|/*c* = *t′* − *R*, (1)

Since the signal propagates at the finite speed *c*, the effect observed at ***x***(*t*′) must originate from a signal emitted by the source at an earlier instant, known as the retarded time, *t′* − *R*, from the corresponding position along its trajectory, ***x****_s_*(*t′* − *R*).

Throughout this work, it is assumed that the source velocity remains lower than the propagation speed of the disturbance, i.e.,

(2) $$\dot{x_{s}}\left(t\right)=\frac{d}{\mathrm{dt}}\left(x_{s}\left(t\right)\right)<c$$

The objective is to determine the delay *R*, defined as the time elapsed between the emission of a disturbance at a previous position along the source trajectory and its arrival, at the observation time *t*′, at a probe moving freely along its own trajectory, with the disturbance propagating at speed *c*.

By expressing this condition mathematically, the propagation delay *R* is obtained as the solution of the following 3D equation:

(3) $${\left|x_{s}\left(\mathrm{t\prime}-R\right)-x\left(\mathrm{t\prime}\right)\right|}^{2}={\left(\mathrm{cR}\right)}^{2}.$$

For a given observation time *t*′, the propagation delay *R* is constrained to the interval:

0 < *R* < *t*′ − *t*_0_. (4)

The solution of the delay equation is obtained by first evaluating the behavior of the governing function at the two endpoints of this interval and subsequently refining the location of the root through a successive interval-reduction procedure.

At the lower bound of the interval, *R* = 0,

(5) $${\left|x_{s}\left(\mathrm{t\prime}-R\right)-x\left(\mathrm{t\prime}\right)\right|}^{2}>{\left(\mathrm{cR}\right)}^{2}\;=\;0$$

At the upper bound, *R* = *t*′ − *t*_0_,

(6) $${\left|x_{s}\left(\mathrm{t\prime}-R\right)-x\left(\mathrm{t\prime}\right)\right|}^{2}<{\left(c(\mathrm{t\prime}-t_{0})\right)}^{2}$$

Therefore, the function defining the delay equation changes sign within the interval (0, *t*′ − *t*_0_), guaranteeing the existence of at least one solution for *R*.

The delay estimation algorithm can be summarized as follows:

1. Select an initial estimate of the delay R within its admissible bounds.
2. Evaluate ${\left|x_{s}\left(t\prime -R\right)-x\left(t\prime \right)\right|}^{2}$.
  - If ${\left|x_{s}\left(t\prime -R\right)-x\left(t\prime \right)\right|}^{2}>{\left(\mathrm{cR}\right)}^{2}$ increase the current estimate of *R*.
  - If ${\left|x_{s}\left(t\prime -R\right)-x\left(t\prime \right)\right|}^{2}<{\left(\mathrm{cR}\right)}^{2}$ decrease the current estimate of *R*.

The sign of the correction therefore depends on the direction of the inequality, whereas the magnitude of the correction is determined by the difference between the two quantities being compared.

After determining the retarded time, the acoustic field produced by a moving source can be evaluated by solving the following inhomogeneous wave equation:

(7) $$\nabla^{2}\psi -\frac{1}{c^{2}}\frac{\partial^{2}\psi }{{\partial t}^{2}}=-4\pi \;s\left(x,t\right),$$

where *c* is the speed of sound and *s* represents the source. The wave equation can be solved by Green’s function method [29,30]. The following expression is the solution of this equation:

(8) $$\psi \left(x,t\right)=\int \frac{s(x^{\prime },t_{r})}{\left|x-x^{\prime }\right|}d^{3}x^{\prime }.$$

A source that emits a constant frequency perturbation for *f* for *t* > 0, moving according to ***x****_s_*(*t*), may be described by the following function:

*s*(***x***,*t*) = *Aδ*(***x*** − ***x****_s_*(*t*))*e*^−*iωt*^*Θ*(*t*), (9)

where the angular frequency is *ω* = 2*πf*, *A* is a constant representing the amplitude of the generated disturbance, and Θ(*t*) = 0 for *t* ≤ 0 and 1 for *t* > 0 is the unit step function which serves to indicate that the source only emits for *t* > 0. Thus, by calculating the volume integral, we obtain the solution of the wave equation for a field created by a moving source, in the form of the Liénard–Wiechert potentials:

(10) $$\psi \left(x,t\right)=\frac{Ae^{\mathrm{i\omega}t_{r}}}{\left|x(t)-x_{s}\left(t_{r}\right)\right|\left[1-\beta_{s}\left(t_{r}\right)\cdot \hat{n}\left(t_{r}\right)\right]}\Theta \left(t_{r}\right),$$

where $\beta_{s}\left(t_{r}\right)=v_{s}(t_{r})/c,\;\;v_{s}\left(t_{r}\right)={\dot{x}}_{s}(t_{r})$ is the velocity of the source at the retarded time instant, and $\hat{n}\left(t_{r}\right)=\left(x(t)-x_{s}\left(t_{r}\right)\right)/\left|x(t)-x_{s}\left(t_{r}\right)\right|$ is the unit vector in the direction between the position of the source at *t_r_* and the position of interest at the instant *t*.

This summarizes the theoretical principles by which the mechanism of sound propagation between moving sources and listeners under free space conditions has been modeled. The details of the programming of these principles are beyond the scope of this paper, but can be found in [24,25,26].

For static configurations, the propagation distance remains constant, and the retarded time can be obtained directly. However, when either the source or the receiver moves, the retarded time becomes a function of the unknown emission time, resulting in an implicit equation. Consequently, the determination of the delay requires solving the corresponding propagation problem for every received sample.

The proposed simulation framework computes the retarded time exactly by considering the trajectories of both the source and the receiver. For the case of linear motions with constant velocity, the problem can be reformulated as a quadratic equation whose physically meaningful solution provides the emission instant associated with each reception time. This approach avoids the approximations commonly employed in discrete-time delay models and guarantees consistency between the simulated geometry and the generated acoustic signals.

Once the emission time has been obtained, the emitted signal can be evaluated at the corresponding instant and subsequently used to calculate the pressure waveform received at each microphone position.

#### 2.3.2. Signal Interpolation

When solving the acoustic propagation problem, it must be considered that the simulation framework operates with discrete-time signals defined at a finite number of sampling instants. This discrete representation applies not only to the emitted acoustic waveforms but also to the trajectories of both sound sources and receivers. Consequently, the determination of the received pressure signals requires two distinct interpolation stages: (1) trajectory interpolation and (2) acoustic-signal interpolation.

The first stage is associated with the computation of the retarded time, *t_r_*. Once the reception instant has been specified, the corresponding emission instant is determined from the propagation equation described in the previous section. Since source and receiver trajectories are available only at discrete sampling instants, the solution is initially bracketed between two consecutive trajectory samples. An interpolation procedure is then applied to obtain the exact source and receiver positions corresponding to the retarded time, allowing an accurate evaluation of the source-to-receiver distance.

The second stage concerns the emitted acoustic waveform. In general, the retarded time does not coincide with the temporal sampling grid of the source signal. Therefore, once the retarded time and the corresponding propagation distance have been determined, the emitted signal must be evaluated through interpolation. This procedure enables the reconstruction of the signal value associated with the exact emission instant, regardless of whether the source waveform is generated analytically or obtained from a recorded audio signal.

The use of interpolation in both stages avoids the quantization errors that would arise from restricting the calculations to the nearest available samples. As a result, the proposed framework provides a physically consistent representation of propagation delays and preserves the temporal accuracy required for sound-intensity estimation, particularly in scenarios involving moving sources or receivers.

#### 2.3.3. Generation of Multichannel Probe Signals

The procedures described in the previous sections are applied independently to each microphone of the virtual acoustic intensity probe. For every reception instant, the corresponding retarded time, source-to-microphone distance, propagation delay, and attenuation factor are computed according to the instantaneous geometry of the measurement configuration. This process yields a pressure waveform for each microphone channel, reproducing the signals that would be acquired by a real probe operating under the same conditions.

For a conventional pressure–pressure (*p*–*p*) probe, the simulation generates two synchronized pressure signals, *p*_1_(*t*) and *p*_2_(*t*), corresponding to the two microphone positions. These signals constitute the primary input data for the acoustic intensity estimation procedures described in Section 2.4. Since both channels are obtained from the same propagation model, the simulator provides complete control over the acoustic field and eliminates the influence of experimental uncertainties such as sensor mismatch, calibration errors, environmental disturbances, or positioning inaccuracies.

The framework is not restricted to a single microphone pair. The same methodology can be applied to an arbitrary number of sensors, allowing the generation of multichannel datasets for more complex probe configurations. In particular, triaxial intensity probes can be modeled by combining three orthogonal microphone pairs, enabling the estimation of the three Cartesian components of the acoustic intensity vector.

The resulting multichannel signals form a coherent and physically consistent representation of the acoustic field and constitute the basis for the analysis of sound-intensity estimation methods under both static and dynamic measurement conditions.

### 2.4. Acoustic Intensity Estimation Methods

To obtain acoustic intensity, it is necessary to know the physical quantities of pressure and particle velocity at the same point. Pressure can be easily obtained using microphones, whereas obtaining particle velocity with acceptable accuracy is more difficult. Both pressure and particle velocity at the same point are indirectly obtained through two microphones separated by a fixed distance ∆*_r_*.

The instantaneous intensity *i_n_*(*t*) at the midpoint between the microphones, in the direction $\vec{n}$ joining the centers of the two microphones, is given by the product of pressure and particle velocity in that direction:

*i_n_*(*t*) = *p*(*t*) *u_n_*(*t*). (11)

The average intensity *I_n_* in the direction $\vec{n}$ is obtained from the instantaneous value:

(12) $$I_{n}=\underset{T\to \infty }{\lim}\frac{1}{T}\int_{0}^{T}i_{n}\left(t\right)\mathrm{dt}.$$

To obtain the two required quantities, the pressures *p*_1_(*t*) and *p*_2_(*t*) measured by the pair of opposing microphones are used. By averaging, the pressure at the midpoint between the two microphones can be expressed as:

(13) $$p\left(t\right)=\frac{p_{1}\left(t\right)+p_{2}\left(t\right)}{2}.$$

Euler’s equation describes the motion of a unit volume of fluid subjected to a pressure gradient, where *ρ* is the air density:

(14) $$\rho \frac{\partial \vec{u}}{\partial t}=\;-\nabla p.$$

The relationship between the particle velocity component in the direction joining the centers of the microphones, *u_n_*(*t*), and the sound pressure, *p*(*t*) is obtained by integrating the Euler equation, resulting in:

(15) $$u_{n}\left(t\right)=-\frac{1}{\rho }\int_{-\infty }^{t}\frac{\partial p\left(t^{\prime }\right)}{\partial n}dt^{\prime }.$$

The determination of acoustic intensity from this configuration is known as the two-microphone method, in which the microphones are placed facing each other at a fixed distance, forming what is known as a *p–p* probe.

If the total intensity of the acoustic field is desired, and not just the component in the probe’s main direction, a three-dimensional probe must be constructed. This is achieved using six microphones aligned in pairs along three orthogonal axes. In this way, the total intensity of the acoustic field will be the vector sum of the intensities of each pair of microphones.

The proposed simulation framework has been designed as a general virtual environment for acoustic intensity measurements rather than as a platform devoted to the evaluation of a particular signal-processing algorithm. Consequently, two complementary acoustic intensity estimation methods have been implemented, representing the two approaches most commonly employed in practical acoustic intensity measurements. Their inclusion enables the simulator to reproduce different measurement procedures within a common physical framework and provides an additional means of assessing the consistency of the simulated pressure signals. Agreement between both methods therefore constitutes an indirect validation of the proposed simulation environment rather than the primary objective of the present work.

#### 2.4.1. Direct Integration Method (ITM)

Knowing the expressions for acoustic pressure and particle velocity [9], the acoustic intensity can be obtained by averaging over a time interval [*t* − *T*, *t*], as:

(16) $$I_{n}=\frac{1}{2\rho {∆}_{r}T}\int_{t-T}^{t}\left.\left\{\left[p_{1}\left(t^{\prime }\right)+p_{2}\left(t^{\prime }\right)\right]\left(\int_{-\infty }^{t^{\prime }}\left[p_{2}\left(t^{\prime \prime }\right)-{\;p}_{1}(t^{\prime \prime })\right]{\mathrm{dt}}^{\prime \prime }\right)\right\}\right.{\mathrm{dt}}^{\prime }.\;$$

If it is necessary to limit the intensity to a specific frequency band, the signals *p*_1_(*t*) and *p*_2_(*t*) must first be filtered. This is the direct integration method. The averaging time, *T*, may be varied depending on the purpose of the measurement. The drawback of this technique is the need to use many simultaneous filters when measurements over different frequency bands are required, as in the case of determining intensity in one-third octave bands.

#### 2.4.2. Cross-Spectrum Method (IFM)

There is another method for calculating average intensity based on operations in the frequency domain. In this approach, the Fourier transforms of the acoustic pressure signals from both microphones, *p*_1_(*t*) and *p*_2_(*t*) are computed; then, the one-sided cross-spectrum of both signals, $G_{p_{1}p_{2}}(f)$, is calculated. Using its imaginary part, the active component of the average acoustic intensity is obtained as follows [17]:

(17) In=−1ρ∆r∫0∞ImGp1p2f2πfdf.

### 2.5. Validation Scenarios

To assess the physical and numerical consistency of the proposed simulation framework, three controlled numerical experiments were designed. These tests examine whether the implementation reproduces the expected physical behaviour under well-defined conditions, including inverse-square distance dependence, frequency-dependent response, angular sensitivity, and dynamic source–probe configurations. Since some of these reference relationships are directly related to the physical principles incorporated into the simulator, these experiments should be interpreted as consistency tests rather than as fully independent validation cases.

#### 2.5.1. Scenario 1: Axial Motion

The first validation scenario was designed to verify that the proposed simulator correctly reproduces the theoretical decay of acoustic intensity with distance in free-field conditions. For this purpose, a stationary point source was placed at a fixed position, while the virtual probe was moved along the propagation axis. The probe remained aligned with the direction of propagation throughout the experiment, ensuring that the measured intensity corresponded to the maximum component of the acoustic intensity vector.

For a monopole source radiating in a homogeneous and lossless medium, the active acoustic intensity is expected to decrease proportionally to the inverse square of the distance from the source. Consequently, the measured intensity should satisfy a power-law relationship of the form:

*I*(*R*) = *K* *R*^−*p*^, (18)

where *R* is the source-to-probe distance, *K* is a proportionality constant, and the theoretical exponent is *p* = 2. The objective of this scenario was therefore to estimate the exponent *p* from the simulated data and compare it with the theoretical prediction.

Acoustic intensity was calculated using both the temporal integration method (ITM) and the frequency-domain method (IFM) for all analyzed one-third-octave bands. The resulting intensity values were fitted to the proposed power-law model in logarithmic scale, allowing the propagation exponent to be estimated and the agreement between the simulated results and the theoretical free-field behavior to be assessed.

#### 2.5.2. Scenario 2: Vertical Motion

The second validation scenario was designed to evaluate the ability of the simulator to reproduce the angular dependence of acoustic intensity measurements. Unlike the axial-motion experiment, in this case the probe was displaced along a vertical trajectory while maintaining a constant horizontal offset with respect to the source. As a result, both the source-to-probe distance and the angle between the propagation direction and the probe axis varied continuously during the measurement process.

The acoustic source was located at position (1, 3, 4), whereas the center of the virtual probe followed the trajectory (6, 3, *z*), with *z* varying from 1 m to 7 m. The horizontal separation between source and probe remained constant at 5 m, while the source-to-probe distance was given by:

(19) $$R(z)=\sqrt{5^{2}+(z-4{)}^{2}}.$$

Consequently, the incidence angle *α* between the direction of propagation and the probe axis changed as a function of the probe position.

Since the virtual probe measures the component of the acoustic intensity vector along its main axis, the measured intensity depends not only on the propagation distance but also on the relative orientation between the source and the probe. For a monopole source radiating in free field, the measured component is expected to follow the combined dependence:

(20) $$I\propto \frac{\cos(\alpha )}{R^{2}}.$$

Therefore, this scenario simultaneously validates the distance attenuation predicted by the propagation model and the geometric projection associated with the directional nature of acoustic intensity measurements.

The simulated intensity values were used to estimate the incidence angle from the measured acoustic intensity. The estimated angles were then compared with the corresponding geometrical angles derived from the known source and probe positions. The comparison was carried out for both ITM and IFM estimators over the complete set of analyzed frequencies. The quality of the reconstruction was assessed using angular error metrics, such as the root-mean-square error (RMSE). In addition, the propagation exponent *p* was treated as an adjustable parameter in order to verify the consistency of the simulated data with the theoretical inverse-square propagation law.

#### 2.5.3. Scenario 3: Probe Rotation

The third validation scenario was designed to assess the directional response of the virtual acoustic intensity probe. In contrast to the previous experiments, the source-to-probe distance remained constant throughout the simulation, while the probe orientation was progressively modified. This configuration isolates the effect of probe directivity from other factors such as propagation loss or variations in source-to-probe distance, allowing a direct comparison between the simulated response and the theoretical angular behavior of a pressure-pressure intensity probe.

The probe was rotated around its geometric center over an angular range extending from 0° to 180°. During this process, the source position and the probe center remained fixed, ensuring that the only varying parameter was the angle *θ* between the probe axis and the direction of acoustic energy propagation. A total of 1200 angular positions were evaluated, providing a high-resolution characterization of the probe response.

For an ideal pressure-pressure intensity probe, the measured active intensity corresponds to the projection of the acoustic intensity vector onto the probe axis. Consequently, the normalized response is expected to follow a cosine law:

(21) $$\frac{I(\theta )}{I_{\max}}=\cos(\theta ),\;$$

where *I*(*θ*) is the intensity measured at orientation *θ* and *I*_max_ is the maximum intensity obtained when the probe is aligned with the propagation direction. This theoretical behavior provides a convenient reference for evaluating the accuracy of the virtual probe implementation.

The experiment was repeated for the complete set of one-third-octave-band frequencies between 25 Hz and 20 kHz. For each frequency, the simulated intensity was normalized with respect to its maximum value and compared with the theoretical cosine response. The agreement was quantified using the root-mean-square error (RMSE), the maximum absolute error, and the correlation coefficient between the simulated and theoretical curves. This analysis makes it possible to determine not only the overall accuracy of the virtual probe but also the frequency range over which the directional response remains consistent with the theoretical model.

#### 2.5.4. Consistency of Intensity Estimation Methods

The three validation scenarios were intentionally designed to evaluate complementary aspects of the proposed simulation framework. The axial-motion experiment focuses on the reproduction of free-field propagation loss, the vertical-motion experiment assesses the combined effects of distance and source-probe geometry, and the probe-rotation experiment validates the directional response of the virtual intensity probe. Together, these scenarios cover the fundamental physical mechanisms involved in acoustic intensity measurements and provide a comprehensive assessment of the consistency of the propagation model, the virtual probe implementation, and the intensity estimation procedures employed in this work.

The present work focuses deliberately on free-field acoustic propagation under controlled conditions. This modeling strategy has been adopted to validate the fundamental physical principles underlying the proposed virtual experimental platform without introducing additional uncertainties associated with environmental effects or instrumentation imperfections. By isolating the propagation model, the virtual probe and the acoustic intensity estimation procedures, the proposed framework provides a reliable basis for assessing the accuracy of the simulated measurements and for interpreting the influence of individual parameters under well-defined conditions.

Once the physical consistency of the proposed framework has been established, the same simulation architecture can be extended to incorporate additional phenomena, including reflections, diffraction, atmospheric absorption, sensor calibration errors, microphone phase mismatch and environmental disturbances. Consequently, the simplifications adopted in the present work should not be regarded as limitations of the proposed methodology, but rather as a deliberate validation strategy that establishes a robust foundation for future developments.

## 3. Results

The assessment of the proposed virtual experimental platform has been designed to assess the main physical and computational components involved in acoustic intensity measurements. Rather than presenting isolated simulation examples, the selected validation scenarios (see Table 2) have been chosen to verify the consistency of the acoustic propagation model, the virtual probe implementation and the acoustic intensity estimation procedures under representative measurement conditions. Each experiment addresses a different aspect of the framework while comparison with analytical solutions provides a quantitative assessment of the simulator accuracy.

Together, these validation scenarios demonstrate that the proposed framework accurately reproduces the physical behavior expected from acoustic intensity measurements while providing sufficient flexibility to investigate more complex experimental situations in future studies.

### 3.1. Axial Motion Results

**Figure 6:**
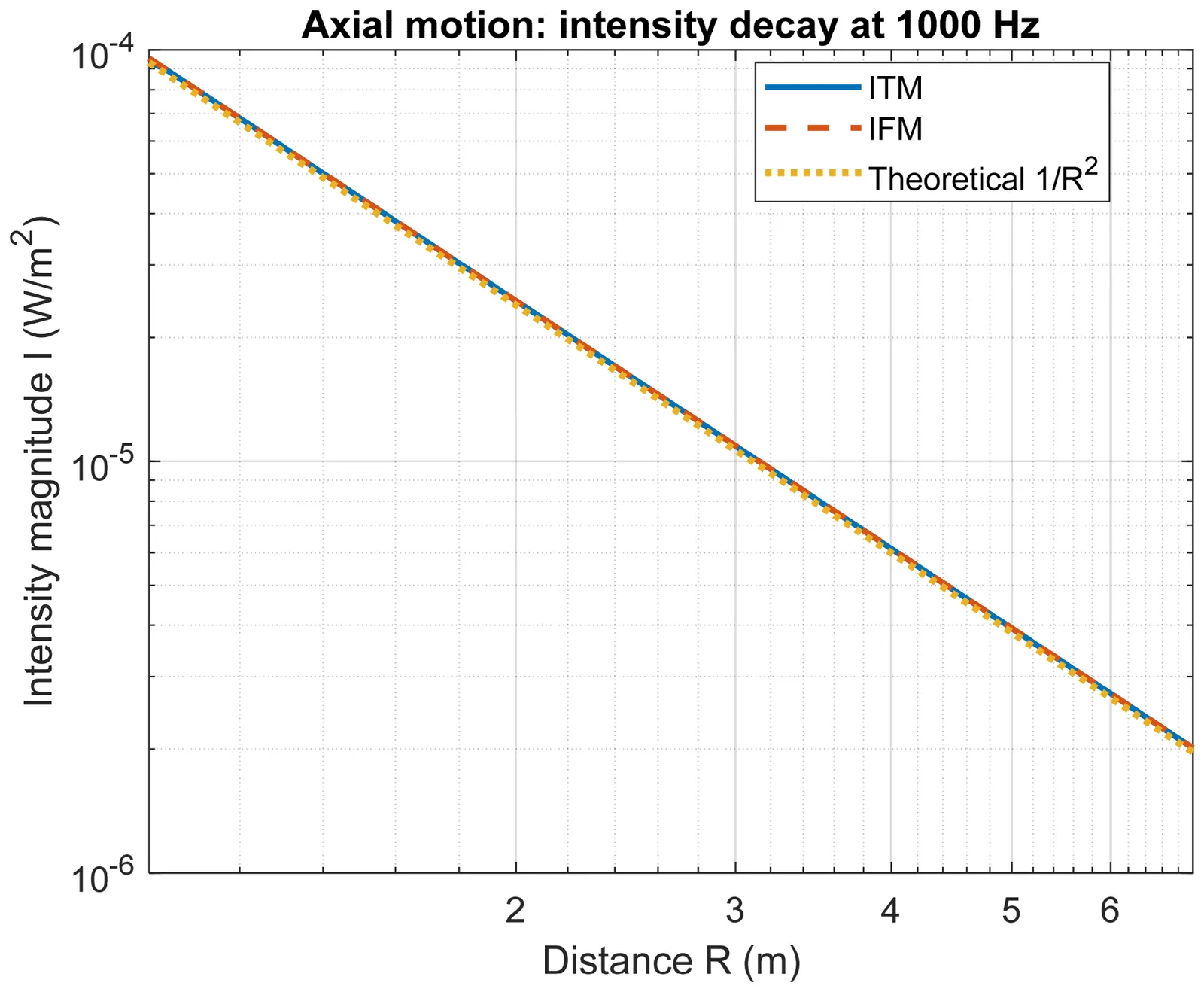
Intensity versus distance for a representative frequency.

This experiment was designed to verify whether the proposed simulation framework reproduces the theoretical free-field attenuation of acoustic intensity with distance. Figure 6 shows the intensity obtained during the axial displacement of the probe together with the theoretical inverse-square law for a representative frequency.

**Figure 7:**
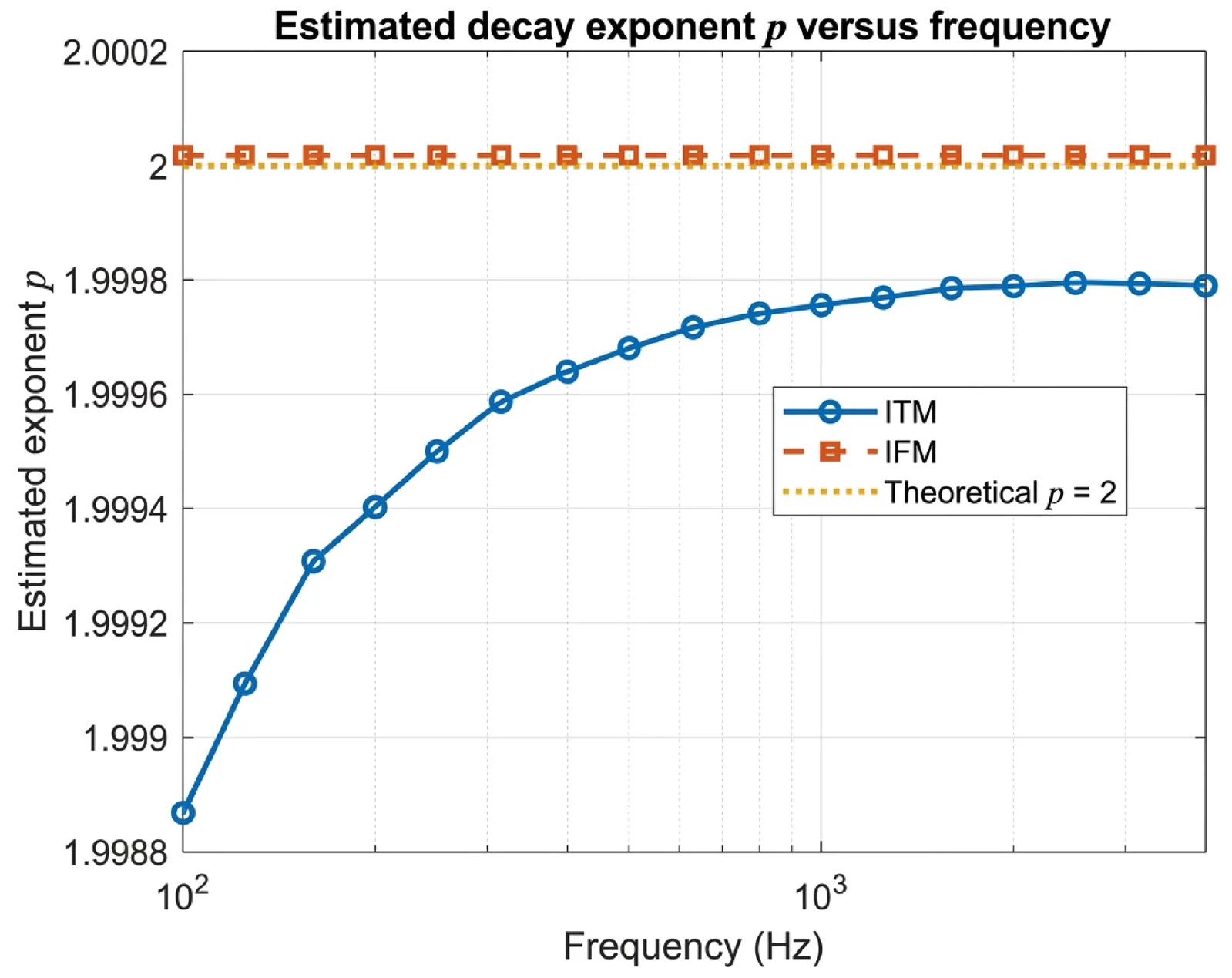
Estimated exponent *p* versus frequency.

A clear decrease in intensity is observed as the source-to-probe distance increases. Both ITM and IFM estimators closely follow the theoretical trend over the complete trajectory. The simulated results remain practically indistinguishable from the analytical prediction, indicating that the propagation model correctly reproduces the expected attenuation behavior. To quantify the agreement with the theoretical model, the simulated intensity values were fitted to the power-law expression *I*(*R*) = *KR*^−*p*^. Figure 7 presents the estimated exponent *p* as a function of frequency for both intensity estimation methods. The estimated exponents remain very close to the theoretical *p* = 2 throughout the frequency range.

**Figure 8:**
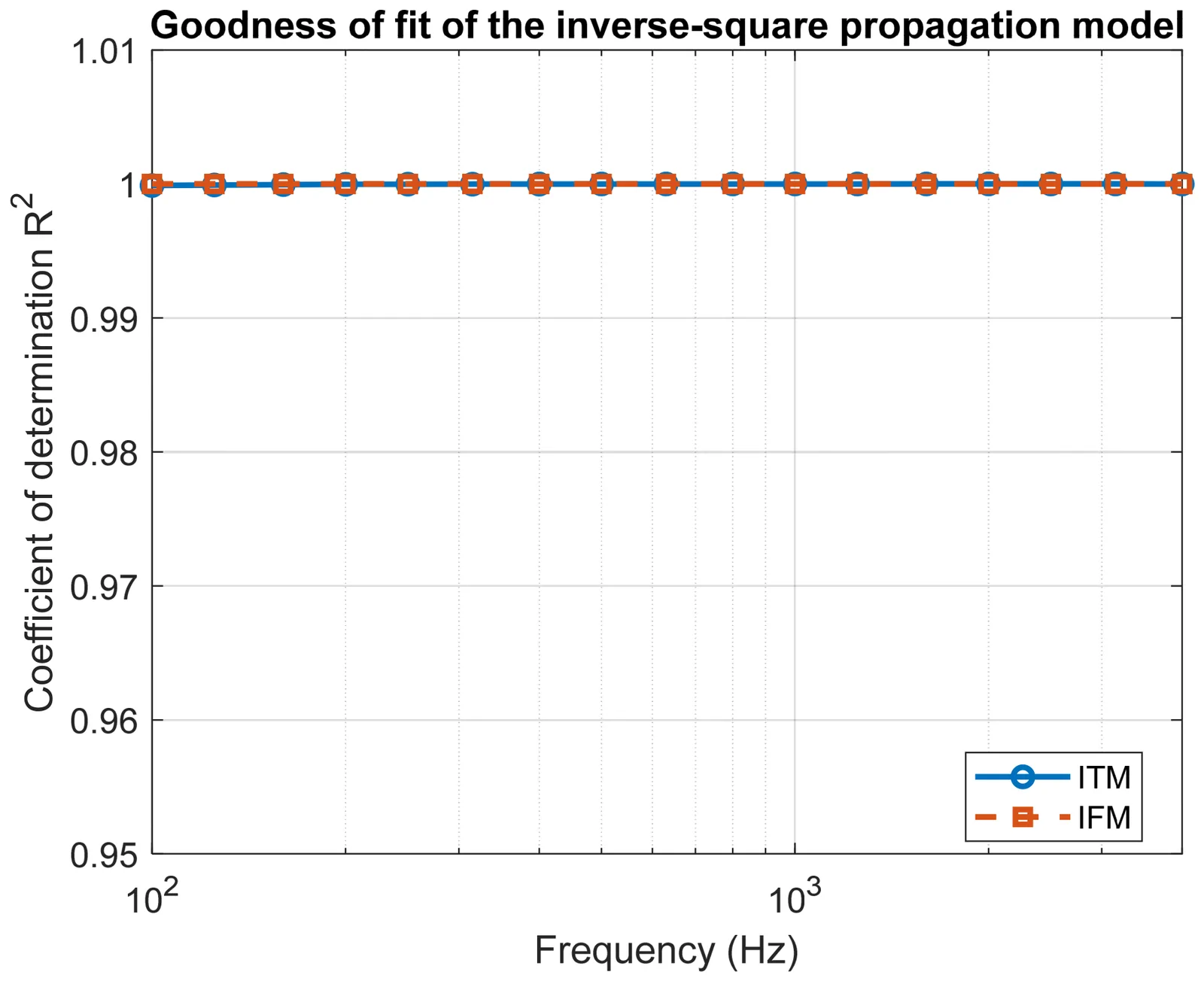
Coefficient of determination *R*^2^.

Figure 8 shows the coefficient of determination obtained for the power-law fitting procedure. Values close to unity were obtained over the complete frequency range, demonstrating that the simulated data exhibit the expected inverse-square dependence with distance.

These results confirm that the proposed simulator accurately reproduces the propagation loss expected for a point source in free field. The agreement between the simulated and theoretical models validates both the retarded-time propagation algorithm and the intensity estimation procedure.

### 3.2. Vertical Motion Results

The second validation scenario evaluates the capability of the simulator to reproduce the combined influence of distance and incidence angle on the measured acoustic intensity. In this experiment, the probe moves vertically while the source remains fixed, so that the measured intensity varies as a consequence of both the changing source-to-probe distance and the angular projection of the intensity vector onto the probe axis.

**Figure 9:**
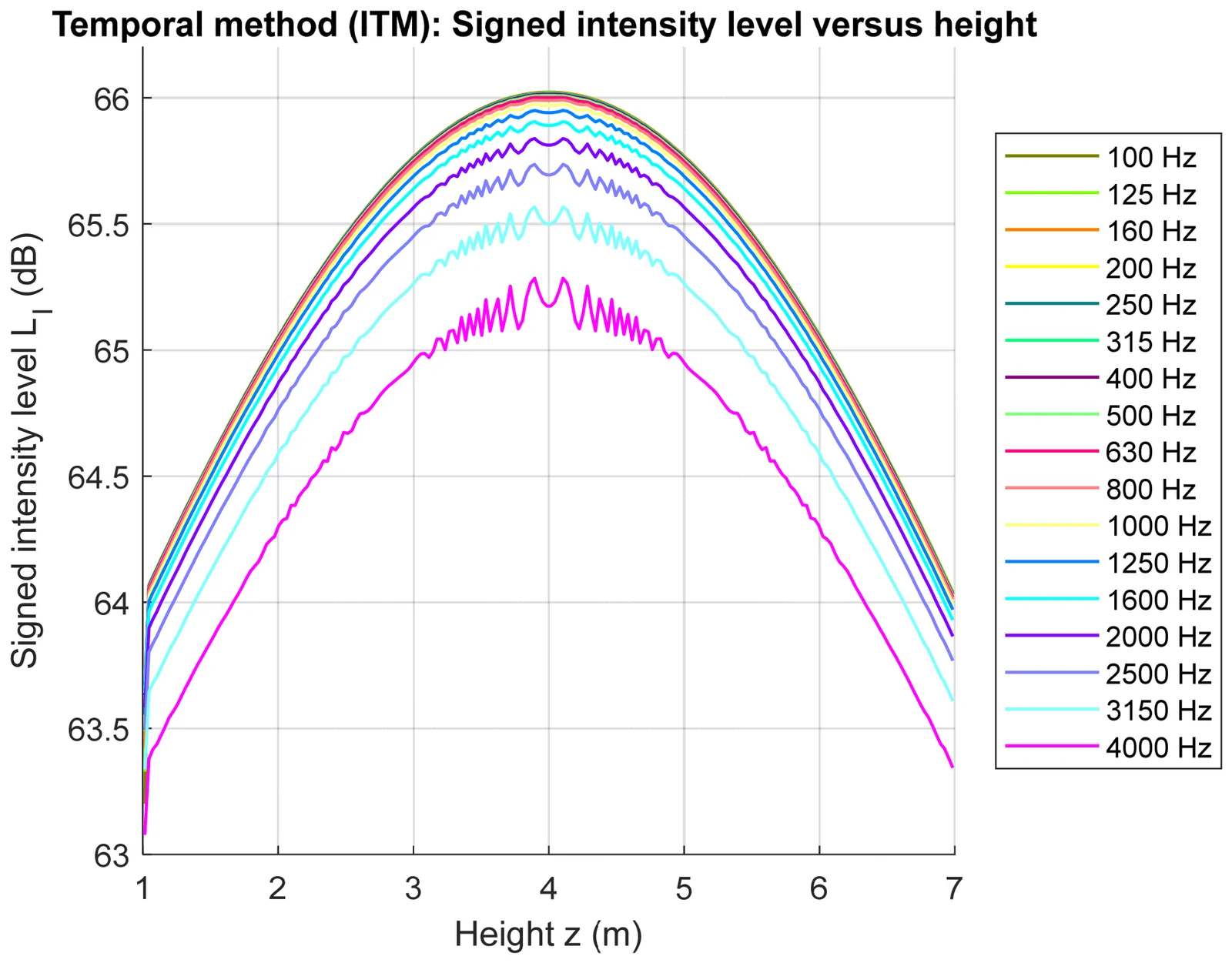
Acoustic intensity level obtained using the frequency-domain method (IFM) as a function of probe height for the vertical-motion validation scenario. $L_{I}\left(\mathrm{dB}\right)=10\log\left(\frac{I}{I_{0}}\right),\;I_{0}=10^{-12}\;W/m^{2}$.

**Figure 10:**
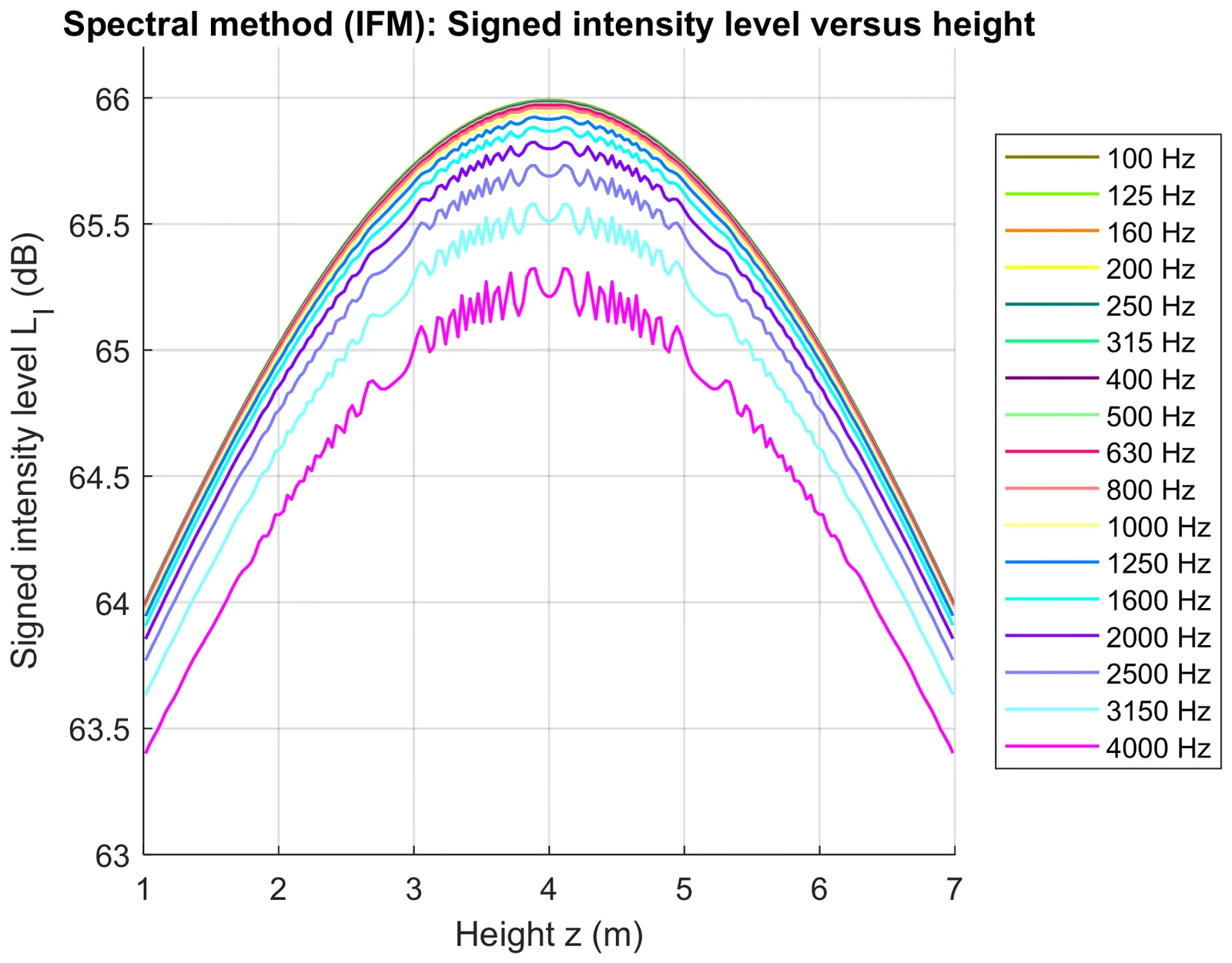
Acoustic intensity level obtained using the time-domain method (ITM) as a function of probe height for the vertical-motion validation scenario. $L_{I}\left(\mathrm{dB}\right)=10\log\left(\frac{I}{I_{0}}\right),\;I_{0}=10^{-12}\;W/m^{2}$.

Figure 9 and Figure 10 illustrate the intensity variation obtained during the vertical-motion experiment using the IFM and ITM estimators, respectively. In both cases, the intensity reaches its maximum value when the probe is located at the same height as the source (*z* = 4 m) and decreases symmetrically as the vertical separation increases. This behavior is consistent with the theoretical dependence of acoustic intensity on both propagation distance and incidence angle. The close agreement between the two estimation methods confirms that the proposed simulator correctly reproduces the geometric effects governing acoustic intensity measurements.

Results are shown for all analyzed one-third-octave-band center frequencies between 100 Hz and 4 kHz.

**Figure 11:**
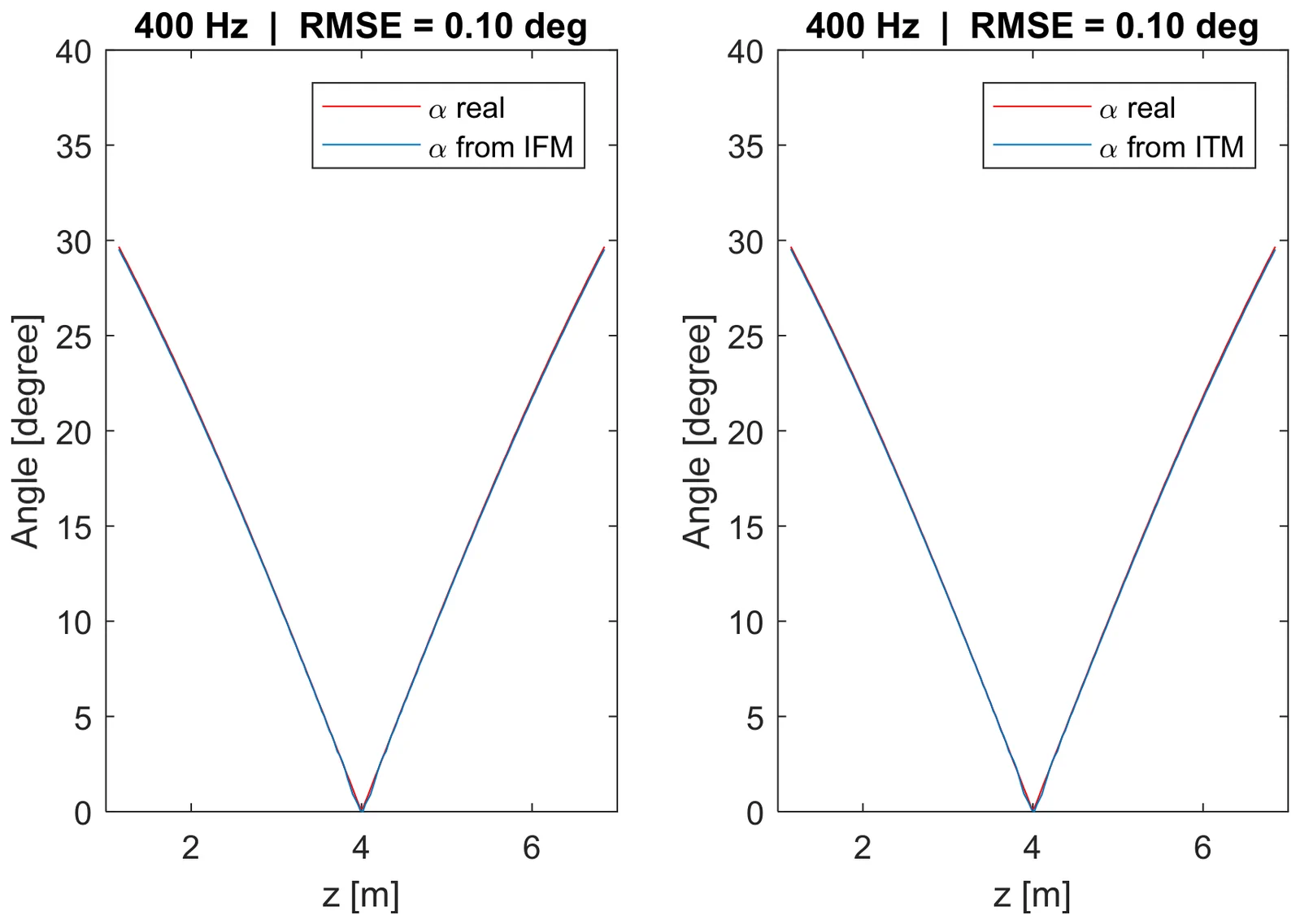
Comparison between the geometrically calculated incidence angle and the angle estimated using ITM and IFM for a single tone of 400 Hz under the vertical-motion scenario.

Figure 11 compares the geometrically calculated incidence angle with the angle estimated from the simulated intensity values for an specific single tone (400 Hz). A close agreement is observed over the complete probe trajectory for both ITM and IFM estimators.

**Figure 12:**
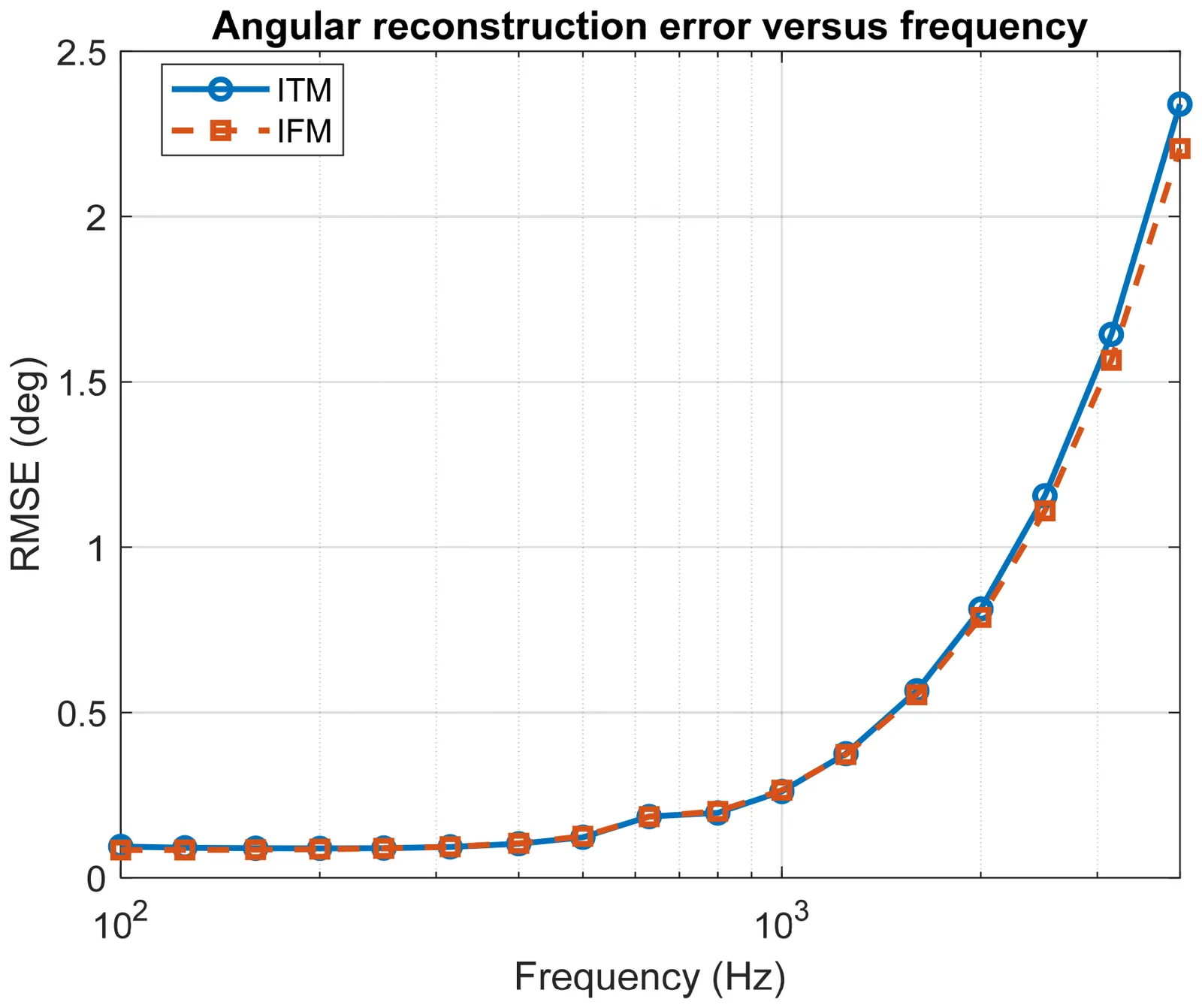
Angular RMSE versus frequency.

To quantify the accuracy of the angular reconstruction, the incidence angle was estimated from the simulated intensity values and compared with the geometrical angle obtained from the known source and probe positions. Figure 12 shows the root-mean-square error of the angular estimation as a function of frequency for both ITM and IFM.

To assess the quality of the angular reconstruction, the root-mean-square error was calculated for all analyzed frequencies. Figure 12 summarizes the obtained results. The angular reconstruction error remains low over most of the analyzed frequency range, indicating that the simulator accurately reproduces the geometric dependence of acoustic intensity measurements.

The propagation exponent was also estimated by minimizing the angular reconstruction error. The optimal values obtained from the simulated data remain close to the theoretical value *p* = 2, providing additional evidence of the physical consistency of the propagation model.

Overall, the vertical-motion experiment validates both the inverse-square propagation law and the directional projection of the acoustic intensity vector, demonstrating that the simulator correctly reproduces the geometric behavior of acoustic intensity measurements. In combination with the axial-motion results, this scenario validates not only the inverse-square propagation law but also the vector projection of acoustic intensity onto the measurement axis of the probe.

### 3.3. Probe Rotation Results

The third validation scenario focuses on the directional response of the virtual acoustic intensity probe. In this experiment, the source-to-probe distance remained constant while the probe orientation was progressively varied between 0° and 180°. This configuration isolates the influence of probe directivity from propagation effects, making it possible to directly compare the simulated measurements with the theoretical cosine response expected for an ideal pressure-pressure intensity probe.

**Figure 13:**
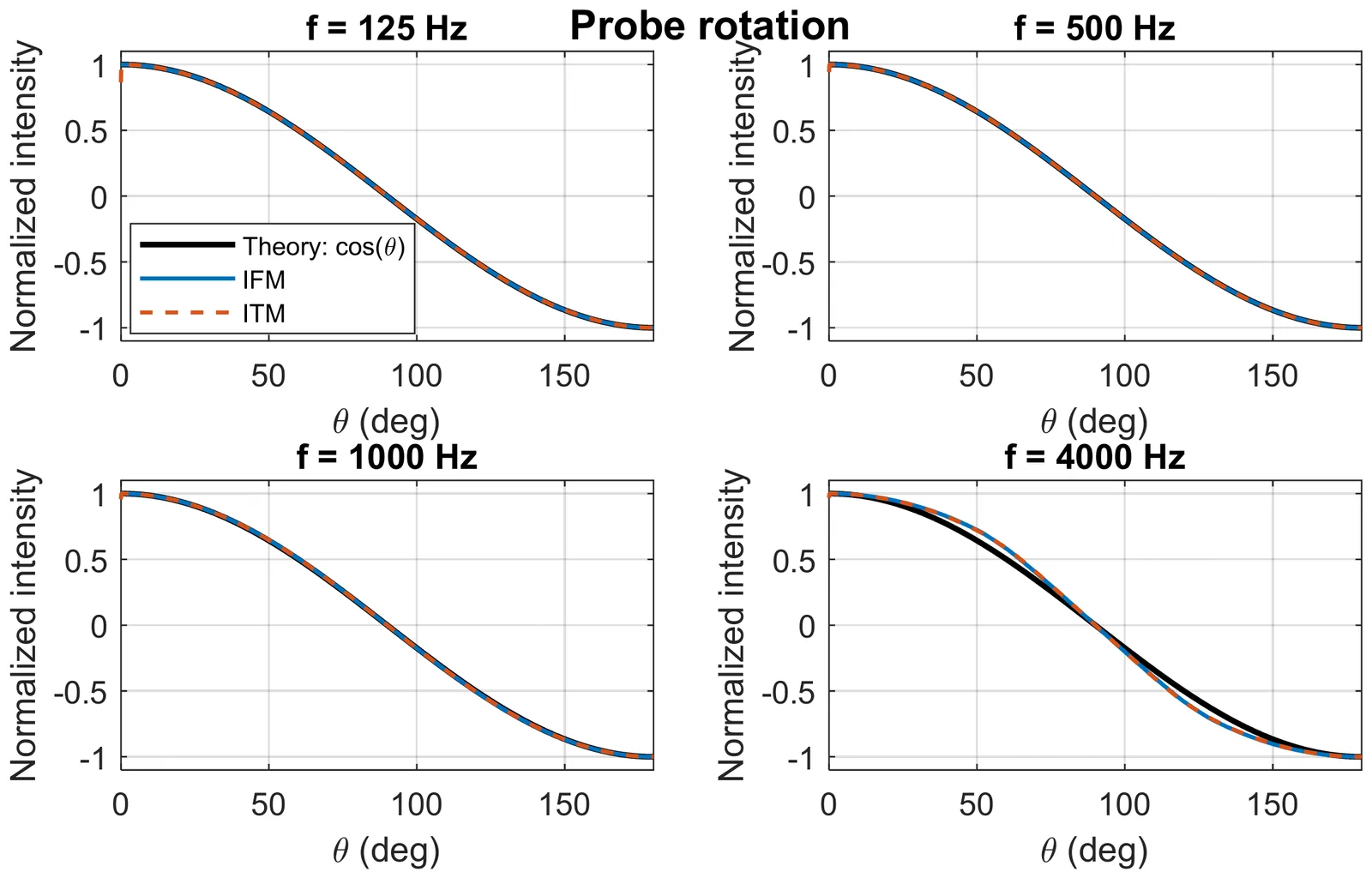
Angular response. Comparison theoretical model, ITM, IFM.

Figure 13 compares the normalized angular responses obtained from the simulations with the theoretical cosine law for several representative frequencies. The simulated curves were normalized with respect to their maximum value in order to facilitate comparison with the theoretical model.

Excellent agreement is observed between the simulated responses and the theoretical cosine dependence throughout the analyzed angular range. Both ITM and IFM accurately reproduce the expected directional behavior of the probe, particularly at low and medium frequencies. The largest deviations occur near the angular positions where the measured intensity approaches zero, where the sensitivity of the estimation process becomes inherently more critical. Nevertheless, the overall agreement remains very high for all analyzed frequencies.

To quantify the agreement between the simulated and theoretical angular responses, the root-mean-square error was calculated for each analyzed frequency.

**Figure 14:**
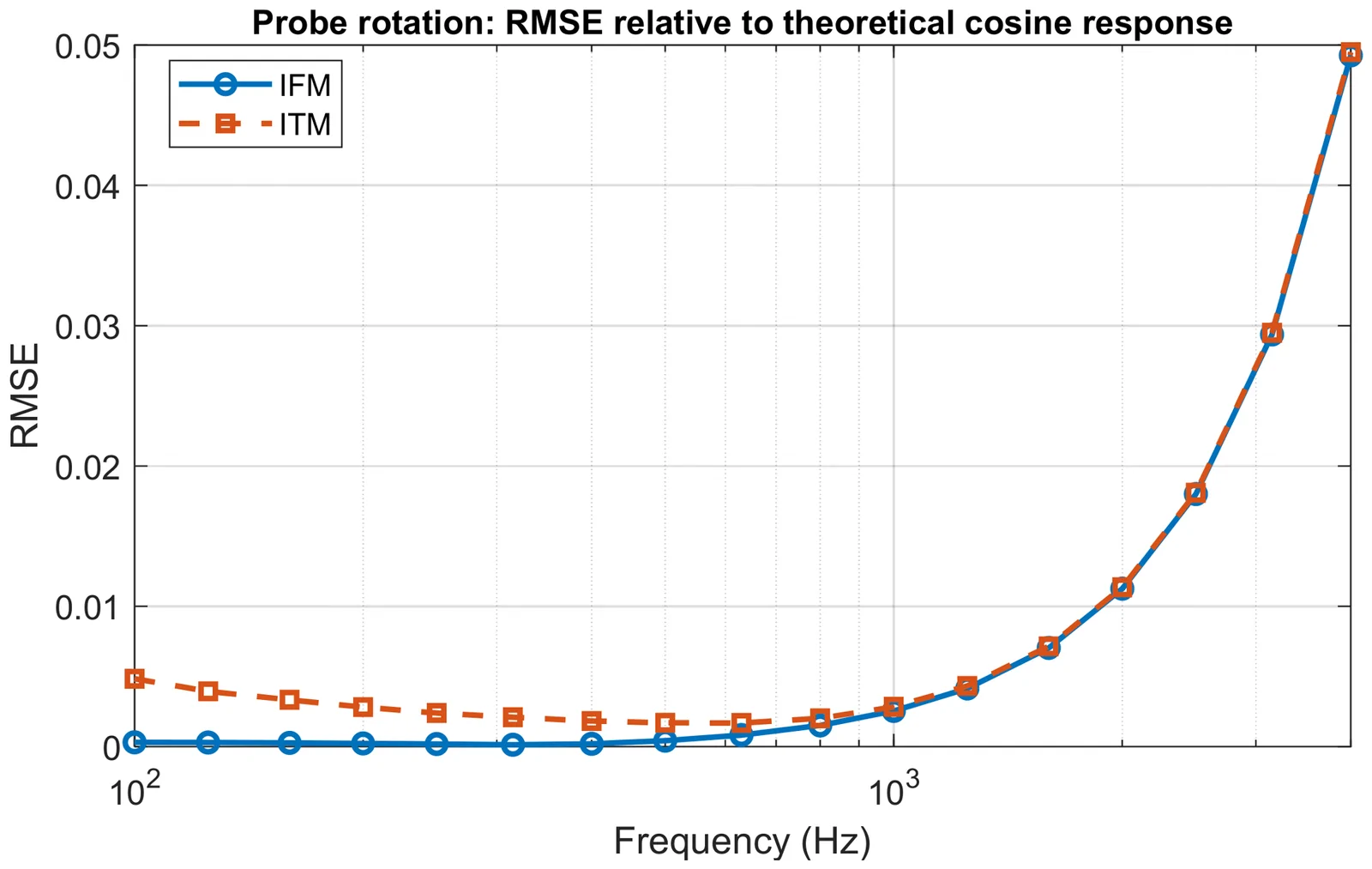
Root-mean-square error between simulated normalized angular response of the rotating probe and the theoretical cosine law as a function of frequency. (For both IFM and ITM estimators).

Figure 14 summarizes the root-mean-square error between the simulated and theoretical angular responses as a function of frequency.

The RMSE remained below 0.05° for all frequencies below 4 kHz, indicating that the virtual probe reproduces the expected directional response with high accuracy. The results obtained using ITM and IFM exhibit very similar trends, confirming the consistency of both estimation procedures. A slight increase in error may be observed as frequency increases, which is consistent with the well-known limitations associated with the finite spacing between the microphones of a pressure-pressure probe.

These results demonstrate that the virtual probe accurately reproduces the directional response of an ideal pressure-pressure intensity probe and remains consistent with the theoretical cosine law over a wide frequency range.

**Figure 15:**
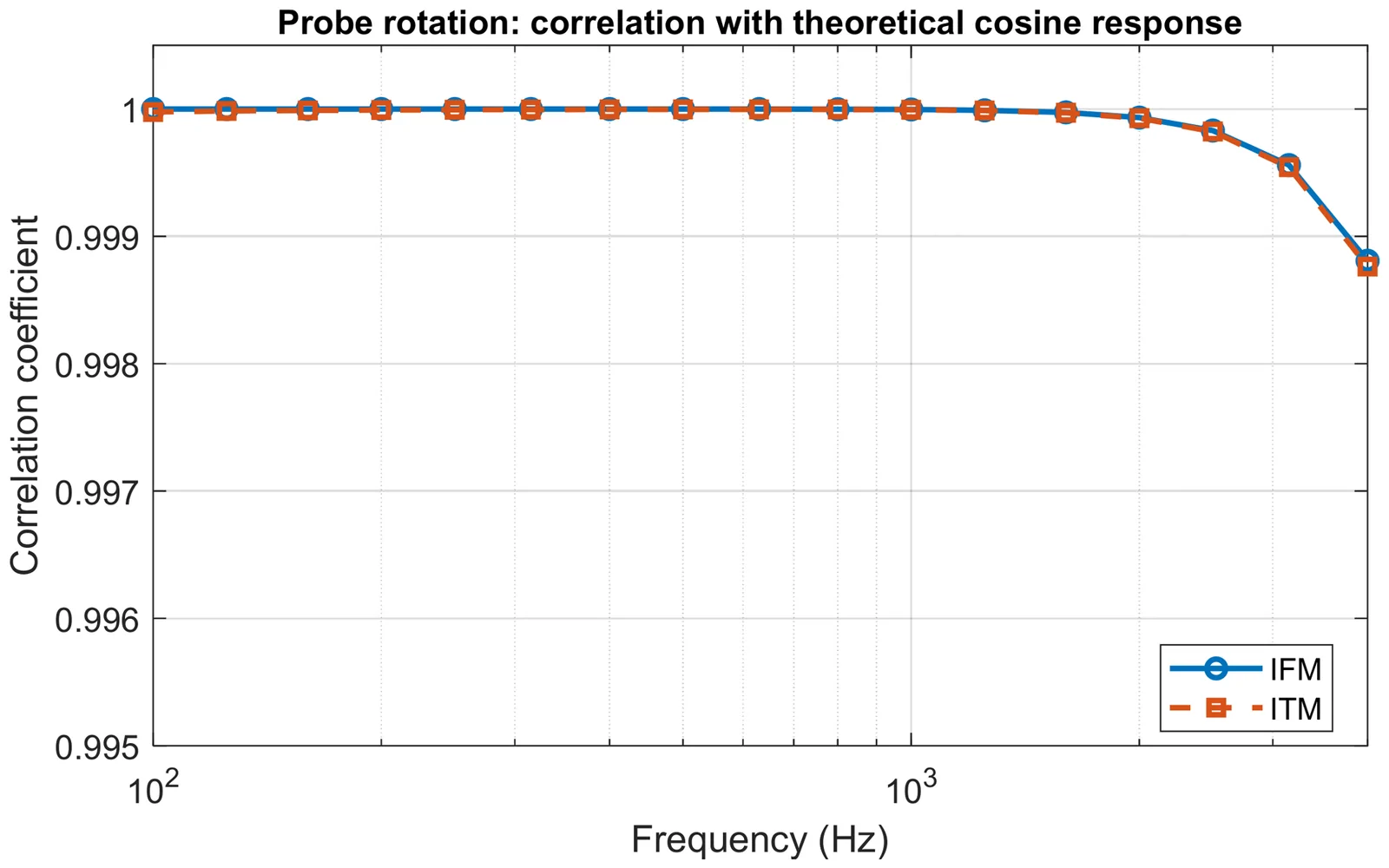
Correlation coefficient between the simulated normalized angular response of the rotating probe and the theoretical law.

Figure 15 shows the correlation coefficient between the simulated normalized angular response of the rotating probe and the theoretical coside law as a function of frequency. Values close to unity indicate that the virtual probe reproduces the expected directional behavior with high accuracy.

The similar behavior obtained with ITM and IFM further confirms the consistency of both estimation procedures within the proposed simulation framework.

### 3.4. Consistency Between ITM and IFM

Since both ITM and IFM estimators are available in the simulator, their consistency was also verified. Previous works [31] demonstrated a high level of agreement between both approaches for a probe spacing of 12 mm over most of the analyzed frequency range.

### 3.5. Summary of Validation Results

To facilitate comparison between the different validation scenarios, Table 3 provides a concise summary of the main quantitative results obtained and their agreement with the corresponding analytical reference solution.

## 4. Discussion

### 4.1. Validation of the Propagation Model

The first validation scenario demonstrated that the proposed framework accurately reproduces the free-field attenuation expected for a monopole source. The estimated propagation exponents remained very close to the theoretical value *p* = 2, while the corresponding coefficients of determination were close to unity throughout the analyzed frequency range. These results indicate that the retarded-time computation, distance estimation procedures, and signal interpolation mechanisms provide a physically consistent representation of acoustic wave propagation.

### 4.2. Validation of Geometrical Effects

The vertical-motion experiment extends the validation beyond simple distance attenuation by incorporating variations in source-probe geometry. In this scenario, the measured intensity depends simultaneously on propagation distance and on the angular projection of the acoustic intensity vector onto the probe axis. The low angular reconstruction errors obtained throughout the analyzed frequency range confirm that the simulator correctly reproduces these geometrical effects. Consequently, the framework is capable of representing realistic measurement situations in which source and receiver are not perfectly aligned.

### 4.3. Validation of Probe Directivity

The probe-rotation experiment provides an independent verification of the directional response of the virtual intensity probe. The excellent agreement observed between the simulated angular responses and the theoretical cosine law confirms that the virtual probe behaves consistently with the theoretical model of an ideal pressure-pressure intensity probe. The low RMSE values and near-unity correlation coefficients obtained across the analyzed frequency range further support the validity of the implemented intensity-estimation procedures.

### 4.4. Scope and Limitations of the Simulation Framework

The present work focuses on the numerical validation of the physical principles governing acoustic intensity measurements against analytical reference solutions under controlled free-field conditions. Consequently, several phenomena commonly encountered in real measurement environments have not been considered, including reflections, diffraction effects, atmospheric absorption, background noise, and environmental disturbances. Moreover, the virtual microphones are idealized and do not incorporate instrumental effects such as microphone mismatch, phase mismatch, calibration errors, or other transducer-related uncertainties. The objective of the proposed framework is not to reproduce every aspect of a specific measurement system, but rather to provide a controlled environment in which the fundamental mechanisms involved in acoustic intensity estimation can be analyzed independently. Therefore, the results presented in this work should be interpreted as a numerical validation of the simulation framework rather than as an experimental validation of a complete acoustic intensity measurement system.

Furthermore, it is worth noting that while the simulation framework mathematically supports a broad frequency range (spanning from 25 Hz to 20 kHz in these scenarios), the actual physical limitations of the simulated *p–p* probe remain governed by its microphone spacing, ∆*_r_*. In practical applications, this spatial separation dictates the operational bandwidth of the sensor. At high frequencies, the measurement accuracy is inherently restricted by the finite-difference approximation error when the acoustic wavelength becomes comparable to the spacer distance. Conversely, at low frequencies, the system’s performance is typically bounded by the phase mismatch between microphones. Although the current idealized free-field model assumes perfect phase matching, identifying these geometric and physical boundaries is essential for properly translating the virtual findings into real-world engineering guidelines.

Despite these simplifications, the simulator offers significant flexibility through its ability to model arbitrary source and probe trajectories, probe rotations, multichannel probe configurations, and different intensity-estimation procedures. This combination of physical consistency and modeling flexibility makes the proposed framework a valuable tool for algorithm development, measurement-system design, and experimental planning.

Taken together, the three validation scenarios demonstrate that the simulator reproduces the three fundamental properties that govern acoustic intensity measurements: free-field propagation loss, geometric projection of the intensity vector, and probe directional response. The consistency observed between theoretical predictions and simulated results suggests that the proposed framework provides a reliable basis for future investigations involving moving sources, moving probes, triaxial intensity measurements, and advanced acoustic sensing applications. Experimental validation using real acoustic intensity probes remains an important next step for assessing the applicability of the framework under practical measurement conditions.

## 5. Conclusions

This work has presented the development and validation of a MATLAB-based simulation framework for acoustic intensity measurements using dual-microphone probes. The proposed model reproduces the generation, propagation, and acquisition of acoustic signals in three-dimensional free-field environments while supporting arbitrary source and probe trajectories, probe rotations, and multichannel probe configurations. The framework incorporates an exact retarded-time formulation together with trajectory and signal interpolation procedures, enabling the simulation of dynamic measurement scenarios involving moving sources and receivers.

Three complementary validation scenarios were designed to assess the physical consistency of the proposed framework. The first scenario verified the inverse-square propagation law through an axial probe displacement. The second scenario evaluated the combined effects of propagation distance and incidence angle during a vertical probe motion. Finally, the third scenario validated the directional response of the virtual probe through controlled rotations and comparison with the theoretical cosine response expected for an ideal pressure-pressure intensity probe.

In all cases, the simulated results exhibited excellent agreement with theoretical predictions. The estimated propagation exponents remained close to the theoretical value expected for free-field propagation, while the angular reconstruction errors and directional-response metrics confirmed the capability of the simulator to reproduce the geometric and vectorial properties of acoustic intensity measurements. Furthermore, both the temporal integration method (ITM) and the frequency-domain method (IFM) produced highly consistent results throughout the analyzed frequency range.

The validation results demonstrate that the proposed simulator accurately reproduces the three fundamental physical mechanisms governing acoustic intensity measurements: propagation loss, directional projection of the acoustic intensity vector, and probe directivity. The main novelty of the proposed framework lies not in the individual formulation of these established acoustic principles, but in their integration within a unified virtual measurement environment that reproduces the complete acoustic intensity measurement chain, from sound propagation and virtual microphone signal generation to intensity estimation and quantitative performance evaluation, while allowing arbitrary motion of both sources and probes. Consequently, the framework provides a reliable environment for investigating the performance of acoustic intensity probes under controlled conditions and for evaluating intensity-estimation algorithms without the uncertainties associated with experimental measurements.

Beyond its validation capabilities, the proposed tool offers considerable flexibility for the simulation of complex measurement scenarios involving moving sources, moving probes, and three-dimensional acoustic fields. These features make it suitable for supporting the design of acoustic measurement systems, the optimization of probe configurations, and the development of advanced signal-processing techniques for acoustic sensing applications. In this context, the framework can serve as a virtual test bench reducing the time and cost associated with repeated measurement campaigns.

The implementation of two independent acoustic intensity estimation methods within the same simulation environment further reinforces the validity of the proposed framework. The close agreement obtained between the estimated intensity values and the theoretical reference demonstrates not only the correctness of the implemented signal-processing procedures but also the physical consistency of the simulated pressure signals from which they are derived.

Despite the promising results, several gaps remain to be addressed in future studies. The present framework deliberately considers controlled free-field propagation and idealized virtual microphones in order to isolate and validate the fundamental mechanisms governing acoustic intensity estimation. Future work will therefore extend the framework to more realistic propagation environments incorporating reflections, diffraction phenomena, atmospheric attenuation, background noise, microphone phase mismatch, calibration errors, and other sensor imperfections. Experimental validation using real acoustic intensity probes constitutes a further important step for quantifying the extent to which the predictions obtained in the virtual environment can be transferred to practical measurement conditions. These developments would progressively bridge the gap between the current controlled simulation framework and complex real-world acoustic measurement scenarios.

## Figures and Tables

**Table 1:** Comparison of the main capabilities of previous simulation approaches and the proposed framework.

Capability	Laville & Nicolas (1992) [22]	Van Ophem et al. (2019) [27]	Barbisan et al. (2026) [25]	Proposed Framework
Acoustic field simulation	✓	✓	✓	✓
Acoustic intensity estimation	✓	✓	—	✓
Virtual dual-microphone *p*–*p* intensity measurement	✓	✓	—	✓
Moving sound source	—	—	✓	✓
Moving receiver/probe	—	—	✓	✓
Arbitrary 3D source/probe trajectories	—	—	✓	✓
Probe translation and rotation	—	—	✓	✓
Propagation-delay/retarded-time treatment under motion	—	—	✓	✓
ITM/IFM intensity estimation	—/✓	—/✓	—/—	✓/✓

**Table 2:** Numerical validation scenarios, framework components assessed, and corresponding reference solutions.

Validation Scenario	Purpose	Framework Component Evaluated	Reference
Axial displacement of the probe	Verify the correct estimation of acoustic intensity when the probe moves along the source–receiver axis	Propagation model, retarded-time computation and intensity estimation	Analytical acoustic intensity
Vertical displacement of the probe	Assess the influence of probe position on the estimated intensity	Spatial interpolation and virtual probe implementation	Analytical acoustic intensity
Probe rotation	Validate the influence of probe orientation on the measured acoustic intensity	Probe orientation model and microphone positioning	Theoretical directional response
ITM and IFM processing	Verify that different intensity estimation methods provide consistent results when applied to the same simulated pressure signals	Signal-processing module	Analytical solution and mutual agreement

**Table 3:** Summary of the main numerical validation results.

Validation Scenario	Main Quantitative Indicator	Main Result
Axial probe motion	*p*, *R*^2^	*p* close to 2; *R*^2^ close to unity
Vertical probe motion	Angular RMSE, *p*	Low angular RMSE; *p* close to 2
Probe rotation	RMSE, correlation	RMSE < 0.05° below 4 kHz; correlation close to unity
ITM and IFM consistency	Agreement between estimators	Very similar behavior for both methods

## Data Availability

The original data presented in the study are openly available in https://drive.google.com/drive/folders/1rKNsOmZYhpyBIoZ-dHhlKPvgNlebgOFG?usp=drive_link (accessed on 20 July 2026).

## Abbreviations

The following abbreviations are used in this manuscript:

ITM	Intensity Time-domain Measurement
IFM	Intensity Frequency-domain Measurement
